# Fasting in combination with the cocktail Sorafenib:Metformin blunts cellular plasticity and promotes liver cancer cell death via poly-metabolic exhaustion

**DOI:** 10.1007/s13402-024-00966-2

**Published:** 2024-07-11

**Authors:** Juan L. López-Cánovas, Beatriz Naranjo-Martínez, Alberto Diaz-Ruiz

**Affiliations:** 1 Laboratory of Cellular and Molecular Gerontology, Precision Nutrition and Aging Program, Institute IMDEA Food (CEI UAM+CSIC), Crta. de Canto Blanco nº 8, Madrid, E-28049 Spain; 2 CIBER Pathophysiology of Obesity and Nutrition (CIBERobn), Córdoba, Spain

**Keywords:** Liver cancer, Fasting, Sorafenib, Metformin, Energy homeostasis, Proteomics

## Abstract

**Purpose:**

Dual-Interventions targeting glucose and oxidative metabolism are receiving increasing attention in cancer therapy. Sorafenib (S) and Metformin (M), two gold-standards in liver cancer, are known for their mitochondrial inhibitory capacity. Fasting, a glucose-limiting strategy, is also emerging as chemotherapy adjuvant. Herein, we explore the anti-carcinogenic response of nutrient restriction in combination with sorafenib:metformin (NR-S:M).

**Results:**

Our data demonstrates that, independently of liver cancer aggressiveness, fasting synergistically boosts the anti-proliferative effects of S:M co-treatment. Metabolic and Cellular plasticity was determined by the examination of mitochondrial and glycolytic activity, cell cycle modulation, activation of cellular apoptosis, and regulation of key signaling and metabolic enzymes. Under NR-S:M conditions, early apoptotic events and the pro-apoptotic Bcl-xS/Bcl-xL ratio were found increased. NR-S:M induced the highest retention in cellular SubG1 phase, consistent with the presence of DNA fragments from cellular apoptosis. Mitochondrial functionality, Mitochondrial ATP-linked respiration, Maximal respiration and Spare respiratory capacity, were all found blunted under NR-S:M conditions. Basal Glycolysis, Glycolytic reserve, and glycolytic capacity, together with the expression of glycogenic (PKM), gluconeogenic (PCK1 and G6PC3), and glycogenolytic enzymes (PYGL, PGM1, and G6PC3), were also negatively impacted by NR-S:M. Lastly, a TMT-proteomic approach corroborated the synchronization of liver cancer metabolic reprogramming with the activation of molecular pathways to drive a quiescent-like status of energetic-collapse and cellular death.

**Conclusion:**

Altogether, we show that the energy-based polytherapy NR-S:M blunts cellular, metabolic and molecular plasticity of liver cancer. Notwithstanding the in vitro design of this study, it holds a promising therapeutic tool worthy of exploration for this tumor pathology.

**Supplementary Information:**

The online version contains supplementary material available at 10.1007/s13402-024-00966-2.

## Introduction

With 41,260 estimated new cases and 30,520 estimated deaths in 2022, primary liver cancer ranks sixth for incidence and fourth for mortality worldwide [1, 2]. Hepatocellular carcinoma (HCC), the dominant type of liver cancer, accounts for approximately 90% of the diagnosis/cases [1, 3]. Infection by Hepatitis B virus (HBV), infection by Hepatitis C virus (HCV), and chronic consumption of alcohol are among the most prevalent risk factors that initiate and favor HCC [3–5]. Increased rates of chronic metabolic diseases also contribute to HCC [6], with nonalcoholic steatohepatitis (NASH) being the fastest growing cause [5]. For a decade now, the first-line treatment for advanced HCC is still Sorafenib, a well-known inhibitor of the serine–threonine kinases Raf-1 and B-Raf and the tyrosine kinase activities of the vascular endothelial growth factor receptors (VEGFRs) and the platelet-derived growth factor receptor β (PDGFR-β) [3, 7]. Noted adverse effects mediated by Sorafenib include diarrhea, fatigue, hand–foot skin reaction, hypertension or even cardiovascular events [8]. Likewise, HCC progression to a Sorafenib-resistant state has been documented [9]. Thus, there is an urgent need to identify sustainable strategies to either enhance Sorafenib treatment efficacy, reduce HCC progression/mortality, and/or minimize Sorafenib dosage and associated treatment toxicity.

Since cancer cells exhibit dysregulated cellular energetics, which exceptionally depends on exacerbated glycolysis for metabolic fueling (‘Warburg effect’), interventions targeting glucose metabolism are of special relevance [10]. Among them, the drug of choice for type 2 diabetes mellitus (T2DM) patients, the oral-biguanide metformin, is shown to have insulin-sensitizing and anti-hyperglycemic properties [11, 12]. Of note, T2DM constitutes the major risk factor for HCC development in patients with nonalcoholic fatty liver disease (NAFLD) [4, 5, 13]. Reasonably, metformin has been independently associated with decreasing the occurrence of HCC and liver-related deaths, likely by the activation of the AMPK pathway [14–18]. Reduction of plasma insulin levels, inhibition of mitochondrial respiration, prevention of protein synthesis, cellular proliferation and angiogenesis, induction of cellular apoptosis, and stimulation of the immune system, are among the other mechanisms also modulated by metformin in liver cancer [19, 20]. Noteworthy, the co-treatment sorafenib:metformin (S:M) is shown to consistently exhibit synergistic effects in preclinical and in vitro models of HCC [21–23], In the clinical practice, retrospective cohort studies have yielded inconclusive findings [24, 25], since S:M is shown to reduces overall survival in HCC patients with pre-existing diagnosis of T2DM, and consequently receiving chronic metformin prior to Sorafenib treatment [26, 27]. Despite these studies, whether simultaneous administration of S:M co-treatment in non-diabetic HCC patients cooperates to promote synergistic effects remain unexplored.

Caloric Restriction (CR), known as a daily reduction of ~ 20–40% calorie intake, and other forms of fasting, are known to impact on glucose metabolism and cellular energetics [28]. Mechanistically, CR-mediated molecular benefits include cellular reprograming of metabolism favoring oxidative phosphorylation (anti-Warburg effect), reduction of anabolic-sensors and associated-signaling (i.e. AKT, mTOR), enhancement of autophagy and stress resistance, and/or homeostatic restoration of intracellular levels of reactive oxidative species [29–31]. In the clinical practice, implementation of fasting as adjuvant therapy potentiates chemotherapy-anticancer effects [32], reduces chemotherapy-related toxicity [33, 34], and differentially reshapes tumor and host metabolism [35, 36]. In preclinical models of HCC, convincing favorable findings have been also documented when CR is applied [31, 37–40]. Notwithstanding this, standardization of fasting in the clinical practice as an adjuvant therapy to specifically combat liver cancer is still not documented [31, 41]. Of relevance for this work, fasting is shown to independently enhance the antitumoral effects by the combination with metformin alone and with sorafenib alone in chronic liver diseases [42] and in liver cancer [9], respectively. Since the number of clinical trials on poly-therapy combinations is growing, side-by-side comparisons of drug combinations and subsequent comprehension of biological mechanisms that underlie drug synergies constitute a central challenge in the clinical landscape [43] and is addressed by this work. Here, we evaluate whether fasting further potentiates the synergistic effects of dual S:M co-treatment is this tumor pathology, which remains not studied. Our results illustrate specific changes with respect to metabolic and bioenergetic status of liver cancer cells, as well as the dynamic response of mitochondrial functionality by the combination of nutrient restriction and S:M (NR-S:M). Likewise, we uncover specific proteomic rearrangements identifying molecular fingerprints with functional relevance in liver cancer.

## Materials and methods

### Reagents

Sorafenib (1, 2.5 and 5 μM) (LC Laboratories, Woburn, USA) was dissolved in DMSO. Moreover, 1,1-Dimethylbiguanide Hydrochloride, 97% (Metformin) (0.5, 1, 5, 10 mM) (Sigma-Aldrich, Madrid, Spain) was dissolved in cell culture medium.

### Cell lines

HepG2, Hep3B and SNU-387 (HB-8065) liver cancer cell lines were purchased from ATCC (Manassas, USA) and cultured as recommended. HepG2, Hep3 liver cancer cells were cultured in Dulbecco’s Modified Eagle’s Medium (DMEM) high glucose 4.5 g/L [19] with L-Glutamine (Thermo Fisher, New York, USA), with 10% fetal bovine serum (FBS, Sigma-Aldrich, Madrid, Spain), 1% antifungal-antibiotic (Penicillin-Streptomycin) (Thermo Fisher) [Ad libitum conditions (AL)]. Moreover, to perform a Nutrient Restriction condition (NR) [44], DMEM low glucose 1 g/L with L-Glutamine, with 2% FBS, 1% Penicillin-Streptomycin was used. SNU-387 liver cancer cell line was cultured in Roswell Park Memorial Institute medium (RPMI-1640, Thermo Fisher), with 10% FBS, 1% Penicillin-Streptomycin and 0.5% Glutamine (Thermo Fisher). In Nutrient Restriction condition, SNU-387 cells were cultured in RPMI-1640 with 2% FBS, 1% Penicillin-Streptomycin and 0.5% Glutamine. Cells were maintained at 37 °C and 5% CO2, and periodically tested for mycoplasma contamination.

### Cell proliferation

Cell proliferation was measured by methylthiazolyldiphenyl-tetrazolium bromide (MTT) assay (Sigma-Aldrich) at 48 h. Briefly, 10,000 cells/well were plated in 96-well plates and after 24 h, treated with Sorafenib (1, 2.5 and 5 μM) and Metformin (0.5, 1, 5, 10 mM), and on the day of measurement, 10 μl of MTT diluted in d-PBS (Sigma-Aldrich) were added to the cells and then incubated 3 h at 37 °C. Subsequently, cells were detached with DMSO, and absorbance measured using a microplate reader (Victor Nivo Multimode plate reader, PerkinElmer, Shanghai, China), at 570 nm. In all instances, cells were plated per quadruplicate. Results are expressed as percentage versus control cells [19]. The coefficient of drug interaction (CDI) is calculated as follows: CDI = AB/(A×B). According to the absorbance of each group, AB is the ratio of the combination groups to control group [19]; A or B is the ratio of the single agent group to control group. Thus, CDI value <1, =1 or >1 indicates that the drugs are synergistic, additive, or antagonistic, respectively.

### Flow cytometry analysis of cell apoptosis and cell cycle

Briefly, 150,000 cells were seeded in 6-well plates, and treated with experimental conditions for 48 h. After treatment, Sorafenib (1 μM), Metformin (5 mM), S:M (1 μM-5 mM, respectively), NR, NR-S (1 μM), NR-M (5 mM) and NR-S:M (1 μM-5 mM, respectively), the harvested cells (previously collected by trypsinization) were washed twice in PBS + 2% FBS and resuspended in 250 μL Annexin buffer. To detected cell apoptosis, Annexin V-FITC/PI Apoptosis Detection Kit (Tonbo Biosciences, CA, USA) was used according to the manufacturer’s instructions. The cells were treated with 5 μl APC Annexin V (Tonbo Bioscience) and 10 μl propidium iodide (PI, Sigma-Aldrich), incubating in dark. The data generated by flow cytometry are plotted in two-dimensional dot plots in which PI is represented versus Annexin V. To cell cycle assay, cells in Annexin buffer, were suspend in PBS (total of 1 mL) and collected carefully in Ethanol. Cells were centrifugated and washed twice in PBS + 3% FBS. Finally, cells were resuspended in 2 mL PBS supplemented with 200 μg/ml PI and 100 μg/mL Ribonuclease A (Sigma-Aldrich) and incubating in the dark at least 30 min. Both protocols were analyzed using a BD FACSCelesta™ flow cytometer (BD Biosciences, San Jose, CA).

### Mitochondrial activity and glycolytic capacity test

To determine Mitochondrial activity and Glycolytic capacity in liver cancer cells, Agilent Seahorse XF Cell Mito Stress Test Kit and Agilent Seahorse XF Glycolysis Stress Test Kit, were used respectively, according to the manufacturer’s instructions. An XF96 extracellular flux analyzer (Agilent, CA, USA) was used. Briefly, 10,000 cells were seeded in Agilent Seahorse XF96 Tissue Culture Microplates and treated with experimental conditions for 3 h. For oxygen consumption rate (OCR) (in picomole of O_2_ per minute) measurement, cells were washed and incubated in XF medium containing 0.5 mM sodium pyruvate, 2 mM glutamine, and 0.5 mM D-Glucose for AL cells, and 2 mM glutamine, without sodium pyruvate and D-Glucose for Nutrient restriction (NR) cells. For extracellular acidification rate (ECAR) (in mpH per minute) measurements, cells were incubated in XF medium containing 0.25 mM sodium pyruvate, 2 mM glutamine without D-glucose for AL and NR cells. After calibration of the analyzer, sequential compound injections, including oligomycin A (2 μM), *carbonylcyanide p-trifluoromethoxyphenylhydrazone* (FCCP; 0.75 μM), and Rotanone/antimycin A (0.5 μM), were applied to test mitochondrial respiration. Sequential compound injections, including Glucose (10 mM), Oligomycin A (2 μM), and 2-DG (75 mM), were applied to test glycolytic activity.

### Retrotranscription and qPCR

Total RNA from cell lines was isolated using TRI Reagent (Sigma-Aldrich). RNA extraction was followed by DNase treatment [45]. The amount and purity of RNA recovered were determined using the NanoDrop 2000 spectrophotometer (Thermo Fisher). RNA (1 µg) was reverse transcribed using the iScript™ Advanced cDNA Synthesis Kit (Bio-Rad, California, USA). qPCR was carried out using the GoTaq® qPCR Master Mix (Promega, WI, USA). Primers of the target genes used in the study were specifically designed with the Primer3 software v.0.4.0 (Suppl. Table S1). To control for variations in the efficiency of the retrotranscription reaction, the expression level of each transcript was adjusted by housekeeping (i.e., ACTB expression). Quantifications were made applying the ΔCt method (ΔCt = [Ct of gene of interest − Ct of housekeeping]). In all cases, the housekeeping gene exhibited a stable expression among experimental groups.

### Protein extraction and western blot

Liver cancer cells were processed to analyze protein levels by western blot after 48 h of treatments exposure. Briefly, 150,000 cells were seeded in 6-well plates, and proteins were extracted using RIPA buffer with EDTA (Thermo Fisher) with Phosphatase Inhibitor Cocktail 1, 2 and 3 and PMSF (1:100). Then, proteins were sonicated for 10 s and boiled for 5 min at 95 °C. Proteins were separated by SDS-PAGE and transferred to nitrocellulose membranes (Millipore, Billerica, MA). Membranes were blocked with 5% nonfat dry milk in Tris-buffered saline, 0.05% Tween 20 and incubated overnight with the specific antibodies for Phospho-p44/42 MAPK (Erk1/2) (Thr202/Tyr204) (#9101, Cell Signaling), LC3A/B (#4108, Cell Signaling), Phospho-AMPKα (Thr172) (40H9, #2535, Cell Signaling), AMPKα (#2532, Cell Signaling) with the appropriate secondary antibody, HRP-conjugated goat antirabbit IgG (#7074S, Cell Signaling). Proteins were detected using an enhanced chemiluminescence detection system (LI-COR, Nebraska, USA) with dyed molecular weight markers (Bio-Rad). A densitometry analysis of the bands obtained was carried out with ImageJ software, using β-actin protein levels as normalizing factor (A1978, Sigma).

### TMT labelling proteomics

The proteome of HepG2 cells after 48 h treatment (8 experimental conditions, *n* = 4 biological replicates, total of 32 samples) was analyzed using 2 kits/versions of TMTpro 18plex reagent [46]. Proteomic profiling, including enzymatic hydrolysis, labeling, mass spectrometry, and bioinformatics analysis, was conducted by The Proteomics Department of the National Biotechnology Center (CNB) in Madrid, Spain. A total of 25 μg from each sample were digested with trypsin followed by TMTpro labelling according with the kit recommendations. Peptide identification was performed using a ThermoUltimate 3000 liquid chromatography unit coupled to a Thermo Orbitrap Exploris OE240 mass spectrometer, operating in DDA (data-dependent acquisition) mode. Separation of labeled tryptic peptides occurred on a C18 reverse-phase column with a 75 μm internal diameter and 50 cm length, using a flow rate of 250 nanoliters/minute over a 120-minute gradient. MS1 and MS2 spectra were collected and utilized for searches against the specific Homo sapiens database downloaded from UniprotKB, employing four combined search engines (Mascot, Sequest, Amanda, and MSFragger), and the results were combined into a single metascore. Signal extraction (reporter ions) from each version of the TMT reagent for quantification was carried out using the Proteome Discoverer v2.5 analysis suite. Subsequently, the standard deviation (SD) of quantified peptides identified in at least 3 biological replicates was calculated to minimize variability within each experimental setup, including technical and labeling variations. Statistical significance for pairwise-comparisons was determined using a t-test (FDR < 0.1) to identify Differential Expressed Proteins (DEPs) between defined groups. Thereafter, specific bioinformatic analysis was performed. First, a comprehensive analysis of specific biological pathways was carried out by the Gene Set Enrichment Analysis (GSEA). GSEA was performed by Genepattern (https://www.genepattern.org/) in Reactome. Next, the online bioinformatics tool Metascape was employed to address the relevance of specific DEPs distinguished by NR-S:M condition [47]. Translational relevance of DEPs was further evaluated using the online server GEPIA2 (a Gene Expression Profiling Interactive Analysis) comparing LIHC (Liver hepatocellular carcinoma) tumoral samples with non-tumoral adjacent tissue extracted from The Cancer Genome Atlas (TCGA) database [48].

### Statistical analysis

Data are expressed as mean ± standard error of the mean (SEM), or relative levels compared with the corresponding controls (set at 100%, 1 for qPCR). All experiments were performed at least 4 biological replicates. Data were evaluated for heterogeneity of variance using the Kolmogorov–Smirnov test and, consequently, parametric (Student t) or nonparametric (Mann-Whitney U) and One-way ANOVA tests followed by uncorrected Fischer were implemented. *P*-values lower than 0.05 were considered statistically significant. All statistics analyses were performed using the GraphPad Prism 8.0 software (La Jolla, CA, USA).

## Results

### Nutrient restriction enhances the synergistic antiproliferative effects of Sorafenib:Metformin

**Fig. 1:**
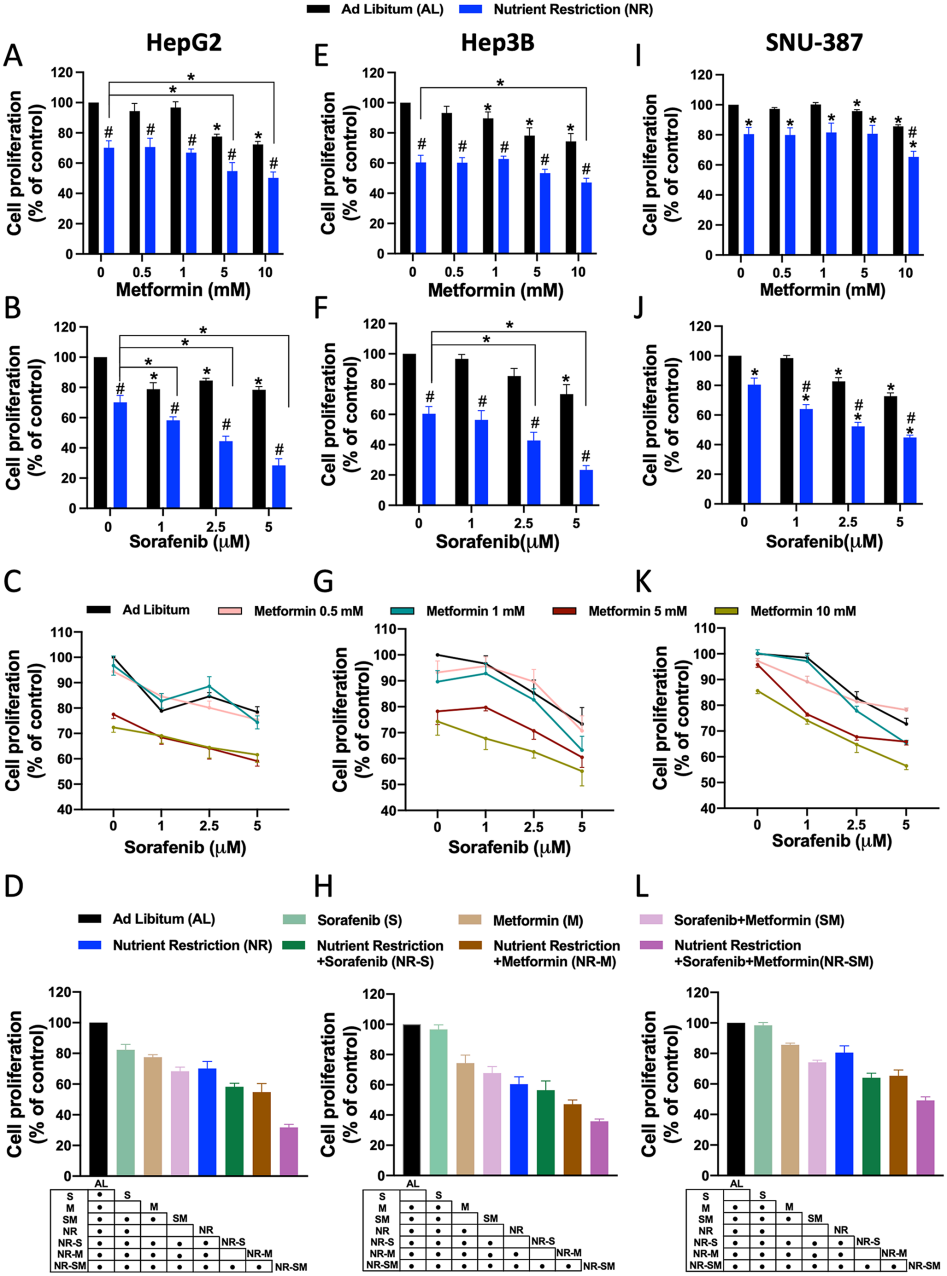
Nutrient Restriction synergistically enhances the antiproliferative effect of Sorafenib:Metformin in three liver cancer cells. (**A-C**) Cell proliferation of HepG2 cells in response of Dose-dependent treatment of **A** Metformin (0.5, 1, 5, 10 mM) **B** Sorafenib (1, 2.5, 5 μM) and **C** co-treatment of Sorafenib (1, 2.5, 5 μM) and Metformin (0.5, 1, 5, 10 mM) compared to non-treated cells, in AL conditions. **D** Cell proliferation of HepG2 cells in response of co-treatment S:M (1 μM-5 mM) in AL and NR conditions compared to non-treated AL cells. (**E-G**) Cell proliferation of Hep3B cells in response of Dose-dependent treatment of **E** Metformin (0.5, 1, 5, 10 mM) **F** Sorafenib (1, 2.5, 5 μM) and **G** co-treatment of Sorafenib (1, 2.5, 5 μM) and Metformin (0.5, 1, 5, 10 mM) compared to non-treated cells, in AL conditions. **H** Cell proliferation of Hep3B cells in response of co-treatment S:M (1 μM-10 mM) in AL and NR conditions compared to non-treated AL cells. (**I-K**) Cell proliferation of SNU-387 cells in response of Dose-dependent treatment of **I** Metformin (0.5, 1, 5, 10 mM) **J** Sorafenib (1, 2.5, 5 μM) and **K** co-treatment of Sorafenib (1, 2.5, 5 μM) and Metformin (0.5, 1, 5, 10 mM) compared to non-treated cells, in AL conditions. **L** Cell proliferation of SNU-387 cells in response of co-treatment S:M (1 μM-10 mM) in AL and NR conditions compared to non-treated AL cells. The data are presented as the means ± SEMs. Student t and One-way ANOVA tests followed by uncorrected Fischer were implemented. Asterisks (*p < 0.05) indicate statistically significant differences vs. AL. Hash symbol (^#^*p* < 0.05) indicate statistically significant differences vs. NR. Points in the table, indicate statistically significant differences (^•^*p* < 0.05)

The capacity of NR to enhance the effects of Sorafenib alone or Metformin alone on cell proliferation was determined by dose-dependent MTT assays in liver cancer-derived HepG2 cells (Fig. 1A, B). Consistent with previous literature [3, 17, 49–51], we observed that Sorafenib (at the doses of 1, 2.5 and 5 μM) and Metformin (at the doses of 5 and 10 mM) significantly inhibit HepG2 viability (Fig. 1A, B, black bars). Likewise, metformin clearly induced the activation of AMPK (Suppl. Figure 1A). Exposure of HepG2 cells to conditions of nutrient scarcity (80% reduction of glucose and serum, herein named nutrient restriction, NR) also reduced cell proliferation by ∼30% and promoted autophagy (Fig. 1C and Suppl. Figure 1A, respectively). Furthermore, the combination of NR with Sorafenib (NR-S) or with Metformin (NR-M) potentiated the antiproliferative effects of Sorafenib or Metformin alone, respectively (Fig. 1A, B, blue bars). We next performed dose-dependent experiments to validate the synergistic effects of Sorafenib:Metformin (S:M) in HepG2 cells (Fig. 1C). To this end, we employed the standard CDI method, which revealed a S:M synergistic effect on cell viability reduction at the doses of S:M (1 μM- 5 mM); S:M (2.5 μM- 5 mM); S:M (5 μM- 5 mM) and S:M (5 μM- 1 mM) (Fig. 1C and Suppl. Figure 1B). Importantly, these results allowed us to select, for each treatment, the lower dose with similar (and significant) effects on cell proliferation, which could represent a potential advantage for the use in the clinic. From now on, experiments in HepG2 cells were performed using the minor CDI dose with synergistic effect [S:M (1 μM- 5 mM)]. At the lower CDI dose, S:M treatment significantly reduced cell viability by ∼30% (Fig. 1D). Remarkably, exposure of cells to NR in combination with the cocktail S:M (NR-S:M) dramatically boosted the effect of S:M, reaching a ∼70% reduction on cell proliferation vs. AL controls, and ∼40% reduction vs. dual S:M therapy (Fig. 1D).

Next, we wanted to decipher the capacity of NR-S:M to reduce cell viability in more aggressive liver cancer cells. To this end, we employed Hep3B cells, which lacks the tumor suppressor p53 gene (Fig. 1E–H), and SNU-387 cells (Fig. 1I–L), in which p53 is mutated [52]. As shown in Fig. 1, Sorafenib alone (Fig. 1E and I, black bars), Metformin alone (Fig. 1F and J, black bars), and NR alone (Fig. 1E and I, blue bars) significantly diminished cell viability in both cell lines. Likewise, implementation of NR potentiated the single effects of Sorafenib alone (Fig. 1E and I, blue bars) or Metformin alone (Fig. 1F and J, blue bars), respectively. Thereafter, dose-dependent experiments were carried out to validate the synergistic effects of S:M. For Hep3B cells, CDI analysis indicated a synergistic S:M antiproliferative effects at the doses of S:M (1 μM- 5 mM, respectively) and S:M (1 μM- 10 mM, respectively) (Fig. 1G and Suppl. Figure 1C), whereas the synergistic S:M doses for SNU-387 cells were defined at S:M (1 μM- 5 mM, respectively) and S:M (1 μM- 10 mM, respectively) (Fig. 1K and Suppl. Figure 1D). At the lower CDI dose selected, S:M cotreatment reduced cellular viability by ∼30% in both cell lines (Fig. 1H and L). Consistent with previous data, addition of NR significantly enhanced this effect reaching levels of reduction ∼70% for Hep3B and ∼50% for SNU-387 cells (Fig. 1H and L). When compared to dual S:M therapy, NR-S:M polytherapy reduced cellular proliferation ∼30% for Hep3B and ∼20% for SNU-387 cells (Fig. 1H and L). Overall, our data reveals that, independently of the aggressivity and molecular properties of liver cancer cells, the synergistic effects of the cocktail S:M on cellular proliferation are further exacerbated by the exposure to short-term starvation.

### Cellular apoptosis is augmented by the triple combination nutrient restriction: Sorafenib:Metformin

**Fig. 2:**
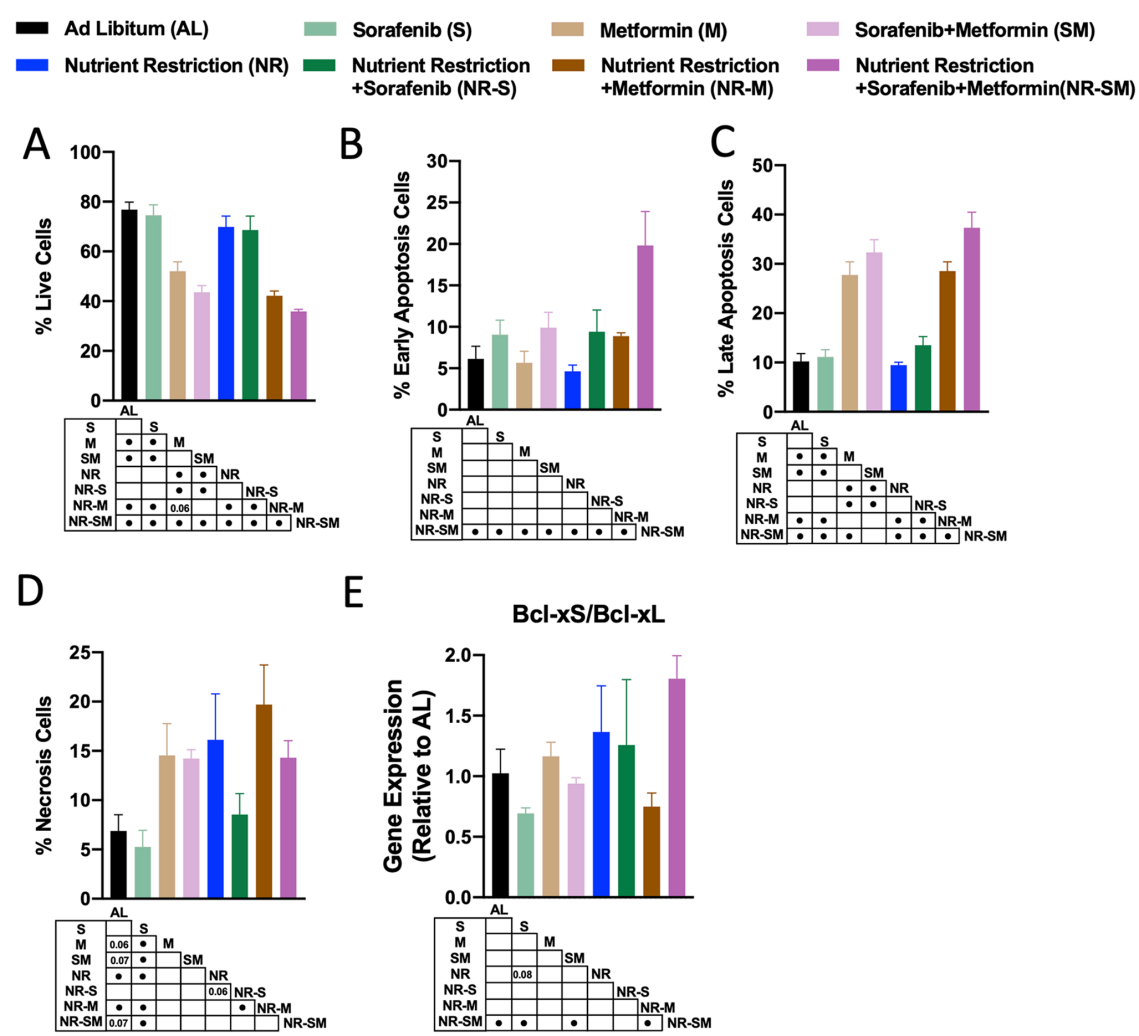
Cellular apoptosis increases by the combination NR-S:M. (**A-D**) Percentage of **A** live cells and cells in **B** Early Apoptosis, **C** Late Apoptosis and **D** Necrosis in response of co-treatment S:M (1 μM–5 mM) in AL and NR conditions compared to non-treated AL cells. **E** Relative expression at mRNA of *Bcl-xS/Bcl-xL* ratio in response of co-treatment S:M (1 μM-5 mM) in AL and NR conditions compared to non-treated AL cells. The data are presented as the means ± SEMs. One-way ANOVA tests followed by uncorrected Fischer were implemented. Points in the table, indicate statistically significant differences (^•^*p* < 0.05)

Cell cytometer-based techniques were carried out in HepG2 to determine if the capacity of NR-S:M to diminish cell proliferation were likely due to an enhancement of cellular apoptosis, a slowness of the cell cycle, or both. The Annexin V-FITC/PI Apoptosis Detection Kit allows the identification of live cells (detection of no labeled annexin/PI at the cell membrane) as well as cells in early apoptosis (detection of labeled annexin V at the cell membrane), late apoptosis (detection of labeled annexinV/PI at the cytosol), or in a necrotic state (detection of labeled annexinV/PI inside the nucleus). As shown in Fig. 2A, metformin alone, but not Sorafenib, significantly reduced the % live cells by ∼30% when compared to AL control cells. The absence of effect for Sorafenib might be explained by the low dose selected [1 μM, which represents the lowest CDI dose with synergistic S:M effects (Fig. 1B)]. Of note, a lower percentage of living cells was reached by the co-treatment S:M vs. Sorafenib alone (Fig. 2A). Analysis of the levels of late apoptotic and necrotic cells revealed a similar situation, with Metformin alone significantly increasing these states by ∼30% and ∼10%, respectively (Fig. 2C, D), and the cocktail S:M further increasing the percentage of cells in late apoptosis (Fig. 2C). Aligned with previous literature, exposure of HepG2 cells to nutrient scarcity did not alter cellular apoptosis, as determined by equal levels of live cells, early and late apoptotic cells (Fig. 2A–C), although a slight induction of necrosis was found (Fig. 2D). Remarkably, NR potentiated the effects of the cocktail of S:M on cellular apoptosis, revealing a strong reduction of % live cells (Fig. 2A, ∼45% reduction) concomitant with a higher induction of early and late apoptosis (Fig. 2B, C, augmentation of ∼15% and ∼30%, respectively, vs. AL. When compared to dual S:M therapy, NR-S:M polytherapy further reduced the % live cells (∼10%; Fig. 2A) and enhanced early apoptotic events (∼10%; Fig. 2B). Escalation of cellular apoptosis by the addition of NR to S:M is in concordance with enhanced reduction of cell proliferation previously exposed (Fig. 1D).

We next determined the mRNA levels of classical markers of apoptosis, two splicing variants of bcl2l1 (Bcl-xL, anti-apoptotic and oncogenic marker [53]; and Bcl-xS, pro-apoptotic tumor suppressor [54]). Unbalance of the ratio Bcl-xS/Bcl-xL was employed as indicator of cellular apoptosis. Likewise, protein levels of the classical anti-apoptotic marker BCL2 was analyzed [55, 56]. As shown in Fig. 2E, exposure of HepG2 cells with Sorafenib alone, Metformin alone or the cocktail S:M unchanged the ratio Bcl-xS/Bcl-xL. Interestingly, addition of NR to the cocktail S:M, but not to single treatments with Sorafenib or Metformin, raised the levels of the apoptotic ratio Bcl-xS/Bcl-xL by ∼2 folds vs. AL controls, and by 2-fold vs. dual S:M therapy (Fig. 2E and Suppl. Figure 2A).

### Cell cycle retention is potentiated by the combination of nutrient restriction with Sorafenib:Metformin co-treatment

**Fig. 3:**
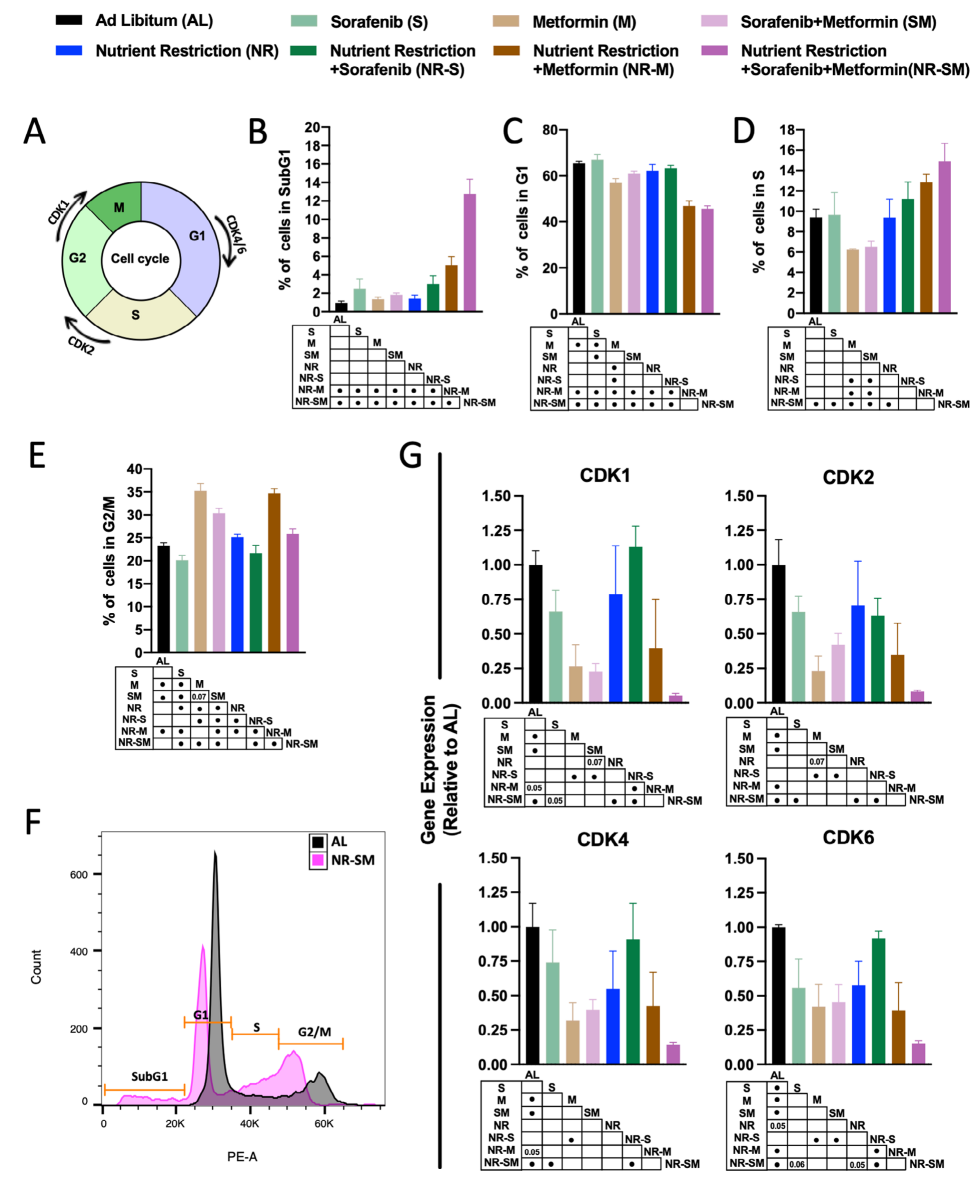
The combination NR-S:M potentiates Cell cycle arrest. **A** Diagram of cell cycle phases (subG1, G1, S and G2/M; and Key cell cycle regulators). (**B-E**) Percentage of cells in **B** SubG1 phase **C** G1 phase **D** S phase and **E** G2/M phase in response of co-treatment S:M (1 μM-5 mM) in AL and NR conditions compared to non-treated AL cells. **F** Representative cell cycle panel in AL condition and NR-S:M. **G** Relative expression at mRNA of *CDK1, CDK2, CDK4, CDK6* in response of co-treatment S:M (1 μM-5 mM) in AL and NR conditions compared to non-treated AL cells. The data are presented as the means ± SEMs. One-way ANOVA tests followed by uncorrected Fischer were implemented. Points in the table, indicate statistically significant differences (^•^*p* < 0.05)

Standard flow cytometer-based analysis of cell cycle phases (subG1, G1, S and G2/M; depicted in Fig. 3A) was then performed. When compared to AL controls, neither Sorafenib alone, Metformin alone, S:M, NR or NR-S altered the percentage of cells in SubG1 (Fig. 3B), whereas SubG1 levels increased ∼5% in NR-M conditions (Fig. 3B). Of note, exposure of cells to NR in combination with S:M further raised cellular levels of SubG1 phase up to ∼12% vs. AL controls, and ∼11% vs. dual S:M therapy (Fig. 3B and F), suggesting that the triple condition NR-S:M would induce the highest levels of DNA fragments [57]. Next, analysis of % cells in G1 phase indicated that treatment with metformin alone, and with the cocktail S:M, but not with Sorafenib, reduced % cells in G1 by ∼10% (*p* value 0.003) and by ∼5% (*p* value 0.09) vs. AL controls. Addition of NR to metformin treatment and to S:M co-treatment, significantly potentiated G1 phase reduction by ∼19% and ∼22%, respectively (Fig. 3C). Analysis of the cellular % in S phase revealed a similar situation. Treatment with metformin alone or with the cocktail S:M, but not with Sorafenib, reduced cellular levels of S phase vs. AL controls by ∼4% in both, although differences did not reach statistical significance (Fig. 3C). Interestingly, a progressive level of retention in S phase was found by the addition of NR to sorafenib, metformin and the cocktail S:M, the latest exhibiting a significant increase of ∼5% when compared to AL, and of ∼10% increase vs. dual S:M therapy (Fig. 3D). Similarly, treatment with metformin alone and with the cocktail S:M, but not with Sorafenib, significantly elevated the % cells in G2/M phase by ∼12% and ∼8%, respectively, vs. AL controls (Fig. 3E). In the context of nutrient scarcity, metformin treatment also enhanced cellular levels of G2/M phase by ∼12% vs. AL controls (Fig. 3E). However, NR-S:M co-treatment reduced G2/M phase levels to those of control cells (Fig. 3E). Overall, our data demonstrate a notorious impact of the triple combination NR-S:M on the modulation of the cellular cycle, which is recapitulated in Fig. 3F, and reinforce the notion of fasting enhancing the synergistic effects of the cocktail S:M (rest of comparisons are included in Suppl. Figure 2B).

Since cyclins are a family of genes involved in cell phases checkpoints, we next analyzed mRNA expression levels of CDK1, CDK2, CDK4 and CDK6. Among other functions, CDK1 is shown to be required to set up the conditions for the transition from S phase to mitosis [58, 59]. CDK4, CDK6 and CDK2 are known to be critical mediators of cellular transition into G1 phase, S phase and G2/M, respectively [60, 61]. As shown in in Fig. 3G, a trend towards lower levels of cyclins vs. AL was found in HepG2 cells after exposure to Sorafenib, reaching statistical significance in the case of CDK6 (Fig. 3G). Remarkably, treatment with metformin alone and with the cocktail S:M strongly inhibited mRNA levels for all cyclin-genes analyzed (Fig. 3G). In the context of nutrient scarcity, a remarkable synergistic effect was found, with NR-S:M showing the lowest levels of expression when compared to every-other condition (reduction of ∼95%, ∼92%, ∼85%, and ∼85% for CDK1, CDK2, CDK4 and CDK6 vs. AL, respectively; and ∼40%, ∼34%, ∼44%, and ∼40% for CDK1, CDK2, CDK4 and CDK6 vs. dual S:M therapy, respectively (Fig. 3G). Next, we investigated the activation of extracellular signal-regulated kinase (ERK) in response to experimental conditions. As shown in the Figure, exposure of HepG2 cells to Sorafenib alone, NR, and/or NR-S interventions, expectedly reduced pERK levels. Contrarily, the presence of metformin (metformin alone, S:M, and NR-S:M treatments) tended to activate pERK. In all cases, differences did not reach statistical significance (Suppl. Figure 2C).

### Glycolytic machinery of liver cancer cells is altered in response to the combination NR-S:M

**Fig. 4:**
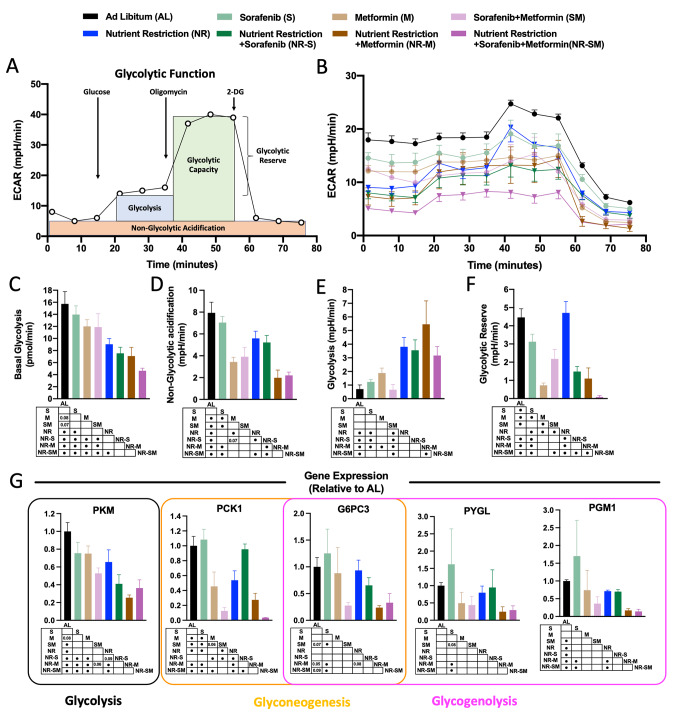
NR-S:M blunts Glycolitic function. **A** Diagram of Seahorse XF Cell Glyco Stress Test Kit. **B** ECAR levels (mpH/min) in response of 3 h of co-treatment S:M (1 μM-5 mM) in AL and NR conditions. (**C-F**) From ECAR, **C** Basal Glycolisis, **D** Non-Glycolytic acidification, **E** Glycolisis, and **F** Glycolytic Reserve in response of co-treatment S:M (1 μM-5 mM) in AL and NR conditions compared to non-treated AL cells. **G** Relative expression at mRNA of *PKM, PCK1, G6PC3, PGYL* and *PGM1* in response of co-treatment S:M (1 μM-5 mM) in AL and NR conditions compared to non-treated AL cells. ECAR: Extracellular acidification rate. The data are presented as the means ± SEMs. One-way ANOVA tests followed by uncorrected Fischer were implemented. Points in the table, indicate statistically significant differences (^•^*p* < 0.05)

**Fig. 5:**
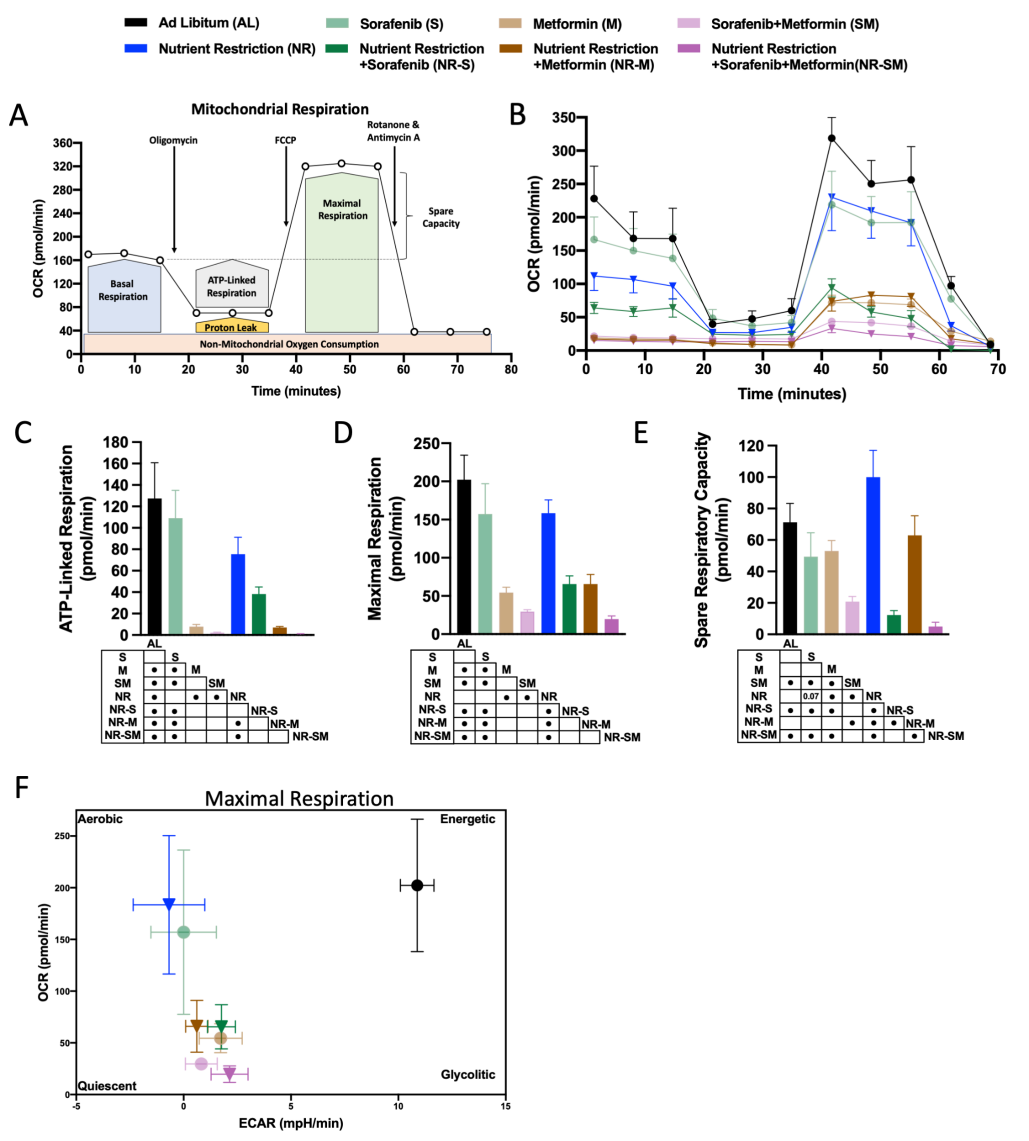
NR-S:M blunts mitochondrial functionality. **A** Diagram of Seahorse XF Cell Mito Stress Test Kit. **B** OCR levels (pmol/min) in response of 3 h of co-treatment S:M (1 μM-5 mM) in AL and NR conditions. (**C-E**) From OCR, **C** ATP-linked Respiration, **D** Maximal Respiration and **E** Spare Respiration Capacity in response of co-treatment S:M (1 μM-5 mM) in AL and NR conditions compared to non-treated AL cells. **F** Metabolic Phenogram Panel of OCR and ECAR rates in Maximal respiration after 3 h of co-treatment S:M (1 μM-5 mM) in AL and NR conditions. OCR: oxygen consumption rate. The data are presented as the means ± SEMs. One-way ANOVA tests followed by uncorrected Fischer were implemented. Points in the table, indicate statistically significant differences (^•^*p* < 0.05)

The glycolytic phenotype of cancer cells has been demonstrated over time [62]. This tumoral characteristic prompted us to decipher the synergistic effect of NR in combination with S:M co-treatment to influence the glycolytic capacity and glycolytic machinery of liver cancer cells. To identify early cellular metabolic responses, evaluation of the extracellular acidification rate (ECAR), determined by Seahorse XF Glycolysis Stress Test Kit (Fig. 4A), together with the analysis of mRNA levels of key enzymes involved in glucose metabolism was carried out in response to 3 h treatment, in which cellular viability is still not compromised (Suppl. Figure 2D). With the exception of the Glycolytic Reserve (GR), capability of a cell to respond to an energetic demand, which was found impaired by Sorafenib treatment (∼33% reduction vs. AL; Fig. 4F), Basal Glycolysis (BsG), Non-glycolytic acidification (nGA) [41], Glycolysis (G) and % Glycolytic Capacity (GC) were unaffected in response to Sorafenib (Fig. 4C–E; Suppl. Figure 2E), in concordance with our previous determinations. Likewise, exposure of HepG2 cells to metformin strongly reduced their GR (∼89% reduction vs. AL; Fig. 4F) and GC (∼50% reduction vs. AL, Suppl. Figure 2E) likely induced by its potent mitochondrial inhibitory capacity (Fig. 5B–E), despite a trend towards increased glycolysis was observed (∼90% increased vs. AL; Fig. 4E). BsG and nGA were also reduced by metformin vs. AL, although differences did not reach statistical significance in BsG (Fig. 4C, D). Of note, co-treatment of HepG2 cells with the cocktail S:M oppositely enhanced GR (Fig. 4F) and oppositely reduced G (Fig. 4E), thus partially reverting the effects mediated by metformin. Interestingly, although S:M co-treatment reduced BsG vs. metformin alone (Fig. 4C), a progressive reduction of these parameters was found by the addition of NR to sorafenib, metformin and the cocktail S:M, the latest exhibiting the lowest levels when compared to every-other condition (∼69% reduction for BsG vs. AL and ∼42% reduction vs. dual S:M therapy; Fig. 4C). NR-S:M polytherapy also reduced nGA when compared to dual S:M therapy (Fig. 4D). Despite these effects, glycolysis (in response to glucose) was found enhanced in all situations in which NR was present (NR-S, ∼400% increased; NR-M, ∼550% increased; NR-S:M,∼300% increased vs. AL controls, respectively; and ∼300% increase vs. dual S:M therapy; Fig. 4E). Strikingly, preservation of the glycolytic reserve, which is dependent on healthful mitochondrial capacity, and was unaffected in response to NR alone, but was strongly impacted by the addition of NR to sorafenib, and synergistically dampened by the triple combination NR-S:M (∼98% reduction vs. AL; ∼90% reduction vs. S:M; Fig. 4F).

We next measured mRNA expression levels of key enzymes involved in (i) glycolysis (PKM, Pyruvate Kinase M1/2), (ii) gluconeogenesis (PCK1, Phosphoenolpyruvate Carboxykinase; and G6PC3, Glucose-6-Phosphatase Catalytic Subunit 3), and (iii) glycogenolysis (PYGL, Glycogen Phosphorylase L; PGM1, Phosphoglucomutase 1; G6PC3). PKM participates in the last step of glycolysis and transfers a phosphoryl group from phosphoenolpyruvate to ADP to generate pyruvate and ATP. As shown in Fig. 4G, all the experimental conditions reduced the levels of PKM vs. AL, although differences did not reach statistical significance for Sorafenib and Metformin treatments (Fig. 4G). Interestingly, the higher reductions were found by the addition of NR to sorafenib, metformin, or the cocktail S:M (Fig. 4G). Thereafter, analysis of gluconeogenic PCK1 and G6PC enzymes, which catalyze 2 of the 3 irreversible steps in the gluconeogenic pathway, was carried out. As shown in Fig. 4G, metformin alone, but not Sorafenib, significantly reduced *PCK1* levels by ∼60% when compared to AL control cells, whereas neither Sorafenib alone nor Metformin alone altered the cellular levels of *G6PC3* gene (Fig. 4G). Exposure of HepG2 to S:M cotreatment provoked a synergistic effect, by means of a higher reduction of both enzymes level (*PCK1*, ∼90% reduction; *G6PC3*, ∼75% reduction vs. AL, Fig. 4G). Of note, the addition of NR to S:M further diminished the expression of *PCK1* (∼99% reduction vs. AL; Fig. 4G), but not that of *G6PC3* (Fig. 4G). Together with *G6PC3*, which catalyzes the hydrolysis of glucose-6-phosphate to glucose and phosphate, glycogenolytic enzymes *PGYL*, which cleavages glycogen to produce glucose-1-phosphate, and *PGM1*, which bidirectionally modulates glucose-1-phosphate and glucose-6-phosphate levels, were measured. When compared to control cells, treatments with Metformin, S:M, NR-M and NR-S:M trended to reduce *PGYL* levels (∼50%, ∼56%, ∼75% and ∼71% reduction, respectively; Fig. 4G). Regarding the *PGM1* levels, all experimental conditions, except for Sorafenib alone and Metformin alone, exhibited inhibition in *PGM1* expression. Notably, the NR-M and the triple condition NR-S:M demonstrated the most significant reductions in expression levels (Fig. 4G).

### Addition of nutrient restriction to the cocktail Sorafenib:Metformin blunts mitochondrial functionality

Several studied have reported that NR, Metformin and Sorafenib induce a reduction of mitochondrial activity [9, 63–67]. Seahorse XF Cell Mito Stress Test Kit (Fig. 5A) was carried out to explore mitochondrial functionality of HepG2 cells in response to 3 h of described treatments. Consistent with previous determinations, the low CDI dose selected for Sorafenib in our studies had a timid influence in all mitochondrial parameters analyzed [basal respiration (BsR), ATP-Linked respiration (ATP-LR), Maximal respiration (MxR), and Spare Respiratory Capacity (SRC), a measure of the ability of the cell to respond to increased energy demand or under stress]; (Fig. 5B–E; Suppl. Figure 2F). In sharp contrast, HepG2 treatment with metformin blunted basal respiration (∼98%, reduction, Fig. 5B; Suppl. Figure 2F) and strongly impacted ATP-Linked and Maximal respiration (∼93% and ∼75% reduction, respectively; Fig. 5B–D) when compared to control cells. Remarkably, a further reduction vs. AL of all three parameters (ATP-LR, ∼98% reduction; MxR, ∼88% reduction; and SRC, ∼71% reduction) was achieved by S:M co-treatment (Fig. 5C–E). In the context of nutrient restriction, a trend towards enhanced SRC was observed for NR alone, despite lower levels of BsR (∼30%), ATP-LR (∼40%), and MxR (∼25%) vs. AL (Fig. 5B–E; Suppl. Figure 2F). Of note, NR potentiated the effects of Sorafenib, by means of significant reduction in all parameters analyzed (BsR, ∼50% reduction; MxR, ∼67% reduction; and SRC, ∼74% reduction) vs. AL. Strikingly, although NR did not exacerbate the effects of metformin, likely by the strong effect of metformin itself, the triple combination NR-S:M further blunted mitochondrial functionality, reaching the lowest levels of ATP-LR, MxR and SRC when compared to every-other condition (∼99% reduction, ∼90% reduction, and ∼93% reduction, respectively, vs. AL controls, respectively; Fig. 5B–E). NR-S:M polytherapy further reduced SRC when compared to dual S:M therapy (∼25% reduction) (Fig. 5B–E). Finally, joint representation of the basal OCR and basal ECAR (Extracellular Acidification Rate) levels provided a snapshot of the bioenergetics profiles of HepG2 after 3 h of treatment. Interestingly, the cocktail S:M, and the addition of NR to S:M co-treatment, compressed the tumoral cells on a quiescent-like state, likely driven by a weakened mitochondrial and glycolytic metabolism (Fig. 5F).

### Molecular rearrangement of the proteome in response to NR-S:M

**Fig. 6:**
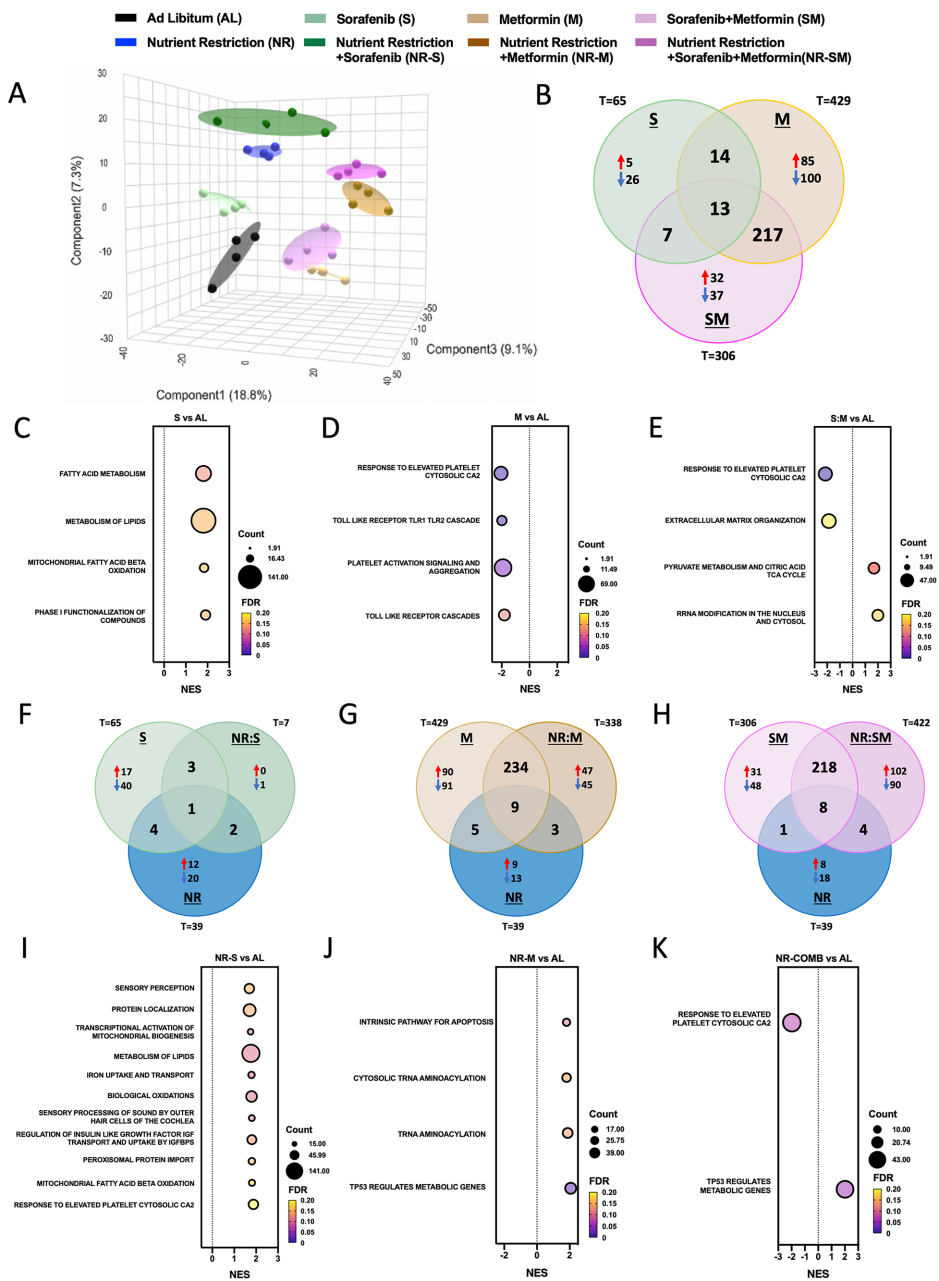
TMT-based proteomic assay elucidates a proteome restructuration triggered by the NR-S:M combination. **A** Partial Least-Squares Discriminant Analysis (PLS-DA) of all protein identified in TMT-based proteomic assay. **B** Venn diagram of Sorafenib, Metformin, and S:M Differential protein vs. AL condition. (**C-E**) Pathways reported by GSEA **C** from Sorafenib, **D** Metformin and **E** S:M vs. AL condition. (**F-H**) Venn diagram of **F** Sorafenib, NR, and NR-S Differential protein vs. AL condition, **G** Metformin, NR, and NR-M Differential protein vs. AL condition, **H** S:M, NR, and NR-S:M Differential protein vs. AL condition. **I** Pathways reported by GSEA from NR-S vs. AL conditions. **J** Pathways reported by GSEA from NR-M vs. AL conditions. **K** Pathways reported by GSEA from NR-S:M vs. AL conditions. GSEA analysis selected by FDR ≤ 0.2

TMT proteomic technology identified 2609 unique proteins that were present in all biological replicates analyzed (Suppl. Table S2). Remarkably, analysis of the global proteome by Partial Least-Squares Discriminant Analysis (PLS-DA) recognized 8 independent and clearly separated clusters (Fig. 6A), suggestive of specific proteomic rearrangements in response to each intervention. Pairwise comparisons were then performed to identify differential expressed proteins (DEPs) (FDR < 0.08); Suppl. Table S3). When compared to AL controls, Sorafenib, Metformin, and S:M treatments regulated 65, 429 and 306 DEPs, respectively (Fig. 6B). Subsequent Gene Set Enrichment Analysis (GSEA; Suppl. Table S4) uncovered that, even at the low dose selected for Sorafenib, molecular pathways (FDR ≤ 0.2) involved in “lipid metabolism” and “beta-oxidation of FA at mitochondria” were enhanced by this treatment (Fig. 6C). On the other hand, exposure of HepG2 cells to metformin downregulated “Toll like receptor cascades” and “Response to elevated Platelet-mediated Ca_2_” and “activation of signaling and aggregation” pathways (Fig. 6D). Interestingly, “Extracellular matrix organization” were found downregulated, whereas “RRNA modification in the nucleous and cytosol” and “Pyruvate Metabolism and TCA cycle” were augmented in response to the cocktail S:M (Fig. 6E).

We next evaluated the effect of NR in the modulation of DEPs and GSEA pathways. In conditions of nutrient scarcity, we found a total of 39 DEPs (Fig. 6F) concomitant with underrepresentation of “Mitochondrial Biogenesis”, “Cristae Formation”, “Pyruvate Metabolism and TCA cycle” as well as “Respiratory Electron Transport and ATP Synthesis” pathways (Suppl. Figure 3A), consistent with a global reduction of the bioenergetic stage. Molecular analysis of the synergistic effect of NR: S revealed the existence of 7 DEPs when compared to AL controls. However, GSEA analysis disclosed the upregulation of multiple pathways, some of them previously activated by Sorafenib alone (Fig. 6C; “lipid metabolism”, “mitochondrial fatty acid beta-oxidation”) and others potentiated by the addition of NR, mainly related to biological oxidation of lipid metabolism (i.e. such as “Regulation of Insulin Like Growth Factors”, “Iron Uptake and Transport”, “Peroxisomal Protein Import”, “Biological Oxidations”, “Transcriptional Activation of Mitochondrial Biogenesis” and “Lipid Metabolism by PPARα” (Fig. 6I). We then analyzed the synergistic effect of NR-M, which recognized 338 DEPs (Fig. 6G) and a notorious potentiation of “intrinsic pathways involved in apoptosis”, “tRNA aminoacylation”, and “TP53 metabolic genes” (Fig. 6J). The absence of regulation of these pathways by either NR alone or Metformin alone emphasizes the potential of the combination of both.

**Fig. 7:**
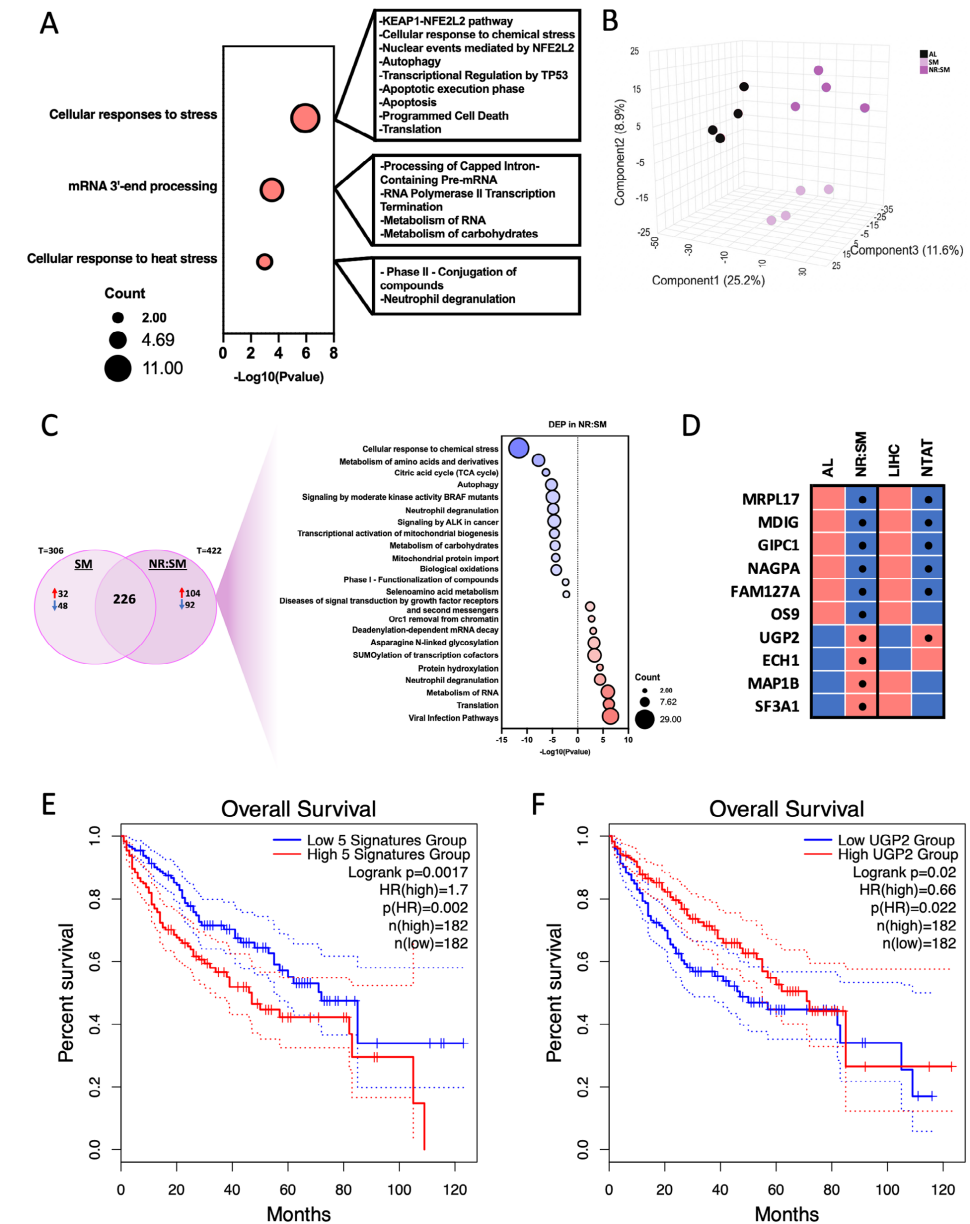
NR-S:M specifically modulates 67 proteins with relevance in Liver cancer. **A** Pathways associated to 67 proteins specifically dysregulated in response to NR-S:M. **B** Partial Least-Squares Discriminant Analysis(PLS-DA) of all protein identified in TMT-based proteomic assay in Groups Al, S:M and NR-S:M. **C** Venn diagram containing differential proteins of NR-S:M vs. S:M condition and Pathways associated to 196 proteins specifically altered in response to NR-S:M (compared only with S:M). **D** Heatmap representation of the Top10 proteins and expression of these proteins in TCGA Liver cancer cohort. **E** Overall Survivalsignature of significant proteins overexpressed in TCGA liver cancer cohort (i.e., MRPL17, MDIG, GIPC1, NAGPA and FAM127A). **F** Overall Survival of UGP2 in TCGA liver cancer cohort.

Last, we deeply explored molecular patterns induced by the addition of nutrient restriction to the cocktail Sorafenib:Metformin. As shown in Fig. 6A, the NR-S:M-mediated proteome displayed unique features that separated from every other intervention. When compared to AL controls, we found 422 DEPs (Fig. 6H). Likewise, “Response to elevated Platelet-mediated Ca_2_” and “TP53 metabolic genes” were found downregulated and augmented, respectively (Fig. 6K), likely indicating a residual activation by either metformin alone and/or NR: M, since metformin and NR induced the highest separation of the proteomes [Fig. 6A, Component 1 (18.8%) and component 3 (9.1%)]. Of note, NR-S:M distinctively altered 67 proteins (FDR < 0.08) not regulated by any other experimental conditions (Suppl. Table S5). Subsequent analysis of the specific NR-S:M signature using the automated meta-analysis metascape tool revealed a significant implication of these proteins in 3 molecular pathways: (1) “Cellular response to stress”, which included, among others, “Apoptotic execution phase”, “Programmed Cell death”, “Autophagy” and “Transcriptional Regulation of TP53”; (2) “mRNA3´-end processing”; and (3) “Cellular response to heat stress” (Fig. 7A). Next, to emphasize in which aspects polytherapy is more effective than dual therapy, pairwise comparisons of their proteome were performed. As shown in Fig. 7B, PLSDA representation of NR-S:M and S:M proteomes clearly classified them in independent clusters. Furthermore, when compared to AL control, we found 196 DEPs exclusively altered by NR-S:M and not by S:M (Fig. 7C). Metascape enrichment analysis uncovered specific NR-S:M down-regulation of molecular pathways whose inhibition have been associated to promote cellular apoptosis and antitumoral effects (“ALK signaling” [68], “Mitochondrial import” [69, 70], and “Metabolism of amino acids and derivates” [71]). Likewise, proteins specifically upregulated by NR-S:M over S:M categorized to molecular activation of genotoxicity, DNA damage and anti-tumoral pathways including “Asparagine N-linked glycosylation” [72], “SUMOylation of transcription cofactors” [73] and “Deadenylation-dependent mRNA Decay” [74]. When viewed together, molecular disruption of these pathways closely aligns with functional data exposed along the manuscript and indicates a distinctive superiority of the polytherapy vs. dual therapy. We next proceeded to investigate the most significant impact induced by NR-S:M by analyzing the Top10 distinctive proteins (6 downregulated, 4 upregulated) that were influenced by NR-S:M (Fig. 7D; Suppl. Table S6). Of note, among the 6 proteins downregulated by NR-S:M in HepG2 cells, 5 of them (MRLP17, MDIG, GIPC1, NAGPA, FAM127A) were statistically and oppositely upregulated in LIHC vs. non-tumoral adjacent tumor (NTAT) (Fig. 7D). Expectedly, these proteins exhibited a strong prediction for overall poor prognosis and survival (Fig. 7E; Hazard ratio = 1.7 and Logrank *p* = 0.0017), thus defining a molecular protein set with translational relevance in LIHC. Likewise, among the 4 proteins upregulated by NR-S:M, UDP-Glucose Pyrophosphorylase 2 (UGP2) was significantly downregulated in LIHC vs. NTAT (Fig. 7D). A higher expression of UGP2 was also associated with enhanced prognosis and overall survival (Fig. 7F; Hazard ratio = 0.66 and Logrank *p* = 0.02).

## Discussion

While performed in vitro, this study shows a polytherapy strategy that utilizes fasting as adjuvant of the co-treatment Sorafenib:Metformin, which has the potential to stimulate clinical advances in the management of HCC. The antitumoral effect of the intervention is tested in three different human models of liver cancer with different degree of genetic complexity and malignancy. We investigated 31 factors with known-metabolic relevance, and we performed a proteomic analysis to decode profiles of bioenergetic dynamics and to decipher molecular patterns provoked in liver cancer cells in response to our combinatory approach. Our data allies with current evidence projected at exploiting reprogramming of cellular metabolism as a promising tool to inhibit cancer growth and invasion [75, 76]. Indeed, integration of glucose-limiting regimens with OXPHOS inhibition constitute an appealing area of research effectively applied for several cancer types [9, 44, 77–79]. Herein, we provide solid data targeting both glycolytic and oxidative metabolism with a triple combination (dietary intervention plus 2 drugs) which induced an energetic crisis that resulted in stimulation of programmed cell death in liver cancer cells.

Proliferative tumor cell exhibit pronounced anabolic appetite, which exceptionally relies on exacerbated glycolysis (‘Warburg effect’). Limitation of glucose (and growth factors) availability by fasting imposes cancer cells to utilize mitochondria, coupled to OXPHOS, to achieve uninterrupted cell proliferation [9, 80]. Herein, we show that fasting impacted glucose metabolism, as determined by diminution of basal glycolysis (Fig. 4C) concomitant with reduction of the glycolytic enzyme PKM (Fig. 4G), and strongly inhibited cellular proliferation (Fig. 1A–J), in concordance with previous studied [81]. However, in response to NR, we found that BsR (∼30%), ATP-LR (∼40%), were all diminished (Fig. 5B–D; Suppl. Figure 2F). Our data is in agreement with the notion that low nutrient availability activates energy-saving mechanisms to maximize energy conservation, which include enhancement of autophagy (Suppl. Figure 1B) and hepatic suppression of mitochondrial metabolism [82, 83]. Reduction of oxygen consumption for the maintenance of energy balance was coordinated at a molecular level by the attenuation of mitochondrial-related pathways (Suppl. Figure 3A, GSEA analysis of the proteome). Noteworthy, a combination of dynamic and quantitative proteomic to determine hepatic proteostasis in response to CR achieved similar results, with CR reducing mitochondrial biogenesis to succeed with cellular fitness [84]. Likewise, reduction of ATP synthesis by fasting has been previously documented [80], in concordance with our data (Suppl. Figure 3B). It should be stated that NR implementation may induce evoke cancer stem-like properties that could favor metastatic re-bounding during periods of refeeding [85–87]. In our case, exposure of cells to NR did not induce cellular apoptosis (Fig. 2, slightly increase of necrosis) nor altered the different phases of cellular mitosis (Fig. 3). In addition, short-starvation followed by a glucose-overload test potentiated glycolysis when compared to non-starved cells (Fig. 4E). Thus, despite fasting being a strong protector against liver diseases (steatosis, liver inflammation, and liver injury), hepatic oncogenesis, and distant metastasis [88, 89], the capability of the above-mentioned cellular shift should be deeply explored in the future.

Fasting has also emerged as a potent adjuvant for standard-of-care drugs, including the multiple-kinase inhibitor Sorafenib [9, 32, 90–92]. Despite Sorafenib main-staying as the first-line treatment for HCC [3, 51], (i) secondary adverse effects (AEs) [8] and (ii) the appearance of sorafenib resistance [9, 93] hampers its efficacy, particularly in the long-term. For the former, dose-reduction is shown to control AEs, lately enhancing treatment response [94]. This conception is in line with our polytherapy approach, which allowed us to in vitro reduce sorafenib dose while maintaining synergistic effects with metformin. Hence, despite the in vitro nature of this study, which implies a limitation, our proposal represents a potential advantage to be considered for further investigation in preclinical and clinical studies, since we mimic major effects achieved in vivo through fasting-based interventions by in vitro utilizing conditions of nutrient scarcity (reduction of glucose and serum, which contains, among other, growth factors). Additionally, it identifies novel molecular events linked to energy-based interventions. Notoriously, at the dose selected, Sorafenib alone was only capable of influence 3 out of the 31 parameters analyzed, which included reduction of cellular proliferation (slight, only in HepG2 cells, Fig. 1B), downregulation of the cell cycle checkpoint gene CDK6 (Fig. 3G), and a reduction of the Glycolytic Reserve (Fig. 4F). The absence of effect on cellular apoptosis and cell cycle arrest when low doses of Sorafenib are used has been previously documented [95, 96]. Unexpectedly, despite the inexistence of major physiological changes, the proteome of HepG2 cells responded to sorafenib by promoting lipid metabolism and fatty acid oxidation pathways (Fig. 6C). In line, a lower dose of Sorafenib has been shown to be efficient at preventing NASH [67]. Beyond this study, the role of a non-toxic Sorafenib dose at controlling lipid and energy metabolism is out of our scope and requires further investigation. Nonetheless, we show that fasting exacerbates sorafenib efficacy, even when the latter is used at a low dose, corroborating previous literature [9, 97]. 11 parameters (out of 31) were regulated by the addition of NR to Sorafenib. Major additive impact of NR-S intervention over NR alone included deterioration of mitochondrial functionality, as determined by reduction of basal, ATP-linked, and maximal respiration, as well as reduction of spare respiratory capacity (Fig. 5), in agreement with other reports [97]. Mitochondrial weakening, together with the fasting-mediated alleviation of sorafenib resistance in liver cancer cells [9], constitute contributing factors favoring our approach. Molecularly, Sorafenib-mediated promotion of lipid metabolism was potentiated by the addition of NR to HepG2 cells, as suggested by the activation of several pathways related to lipid transport and homeostasis such as “Peroxisomal Protein Import” and “Lipid Metabolism by PPAR”. Interestingly, activation of apolipoproteins within these pathways (APOE, APOA1, APOH; Suppl. Figure 4) might also constitute a compensatory mechanism of HepG2 cells to lessen inflammation [98–101]. Likewise, NR-S condition enriched “Biological Oxidations” and “Iron Uptake and Transport” pathways, with specific proteins like HMOX1 (Suppl. Figure 4) known to be activated in response to oxidative stress and cancer cell sensibilization [102, 103].

Metformin, a well-known inhibitor of mitochondrial complex I, constitutes the first-choice treatment for T2DM patients and exhibits an excellent clinical safety profile [14–17, 104]. As previously documented, our data strongly supports the antitumoral and mitochondrial inhibitory capacity of metformin in HCC [15, 18, 19, 105]. Here, 17 parameters (out of 31) were disturbed by the exposure of liver cells to metformin alone. As expected, all indicators of mitochondrial functionality were profoundly affected/diminished (Fig. 5B), as well as the cellular glycolytic reserve (Fig. 4F). Metformin treatment also influenced cell cycle progression by diminishing the expression of all cyclin genes measured and inducing cell cycle arrest at G1 phase and in G2/M phase (Fig. 3C–E), as previously reported [106, 107], increasing apoptosis. Diminution of cellular “Toll like receptor cascades” and the “Response to elevated Platelet-mediated Ca2” and to the “platelet activation of signaling and aggregation” have been also reported as common pathways mediated by metformin [108–110], and with key roles both in the progression of chronic liver disease and HCC [111–113]. Of relevance, metformin exacerbated Sorafenib efficacy. The cocktail S:M significantly altered 17 metabolic factors (out of 31) vs. AL condition, with synergistic reduction of cellular proliferation and spare respiratory capacity over Sorafenib alone and Metformin alone (Fig. 1C, G and K). Overall, demonstration of the negative impact in liver cancer cells by targeting mitochondrial metabolism with S:M co-treatment provides novel insight for the comprehension of the synergetic mechanisms induced by both drugs [114]. In our study, S:M co-treatment downregulated “Extracellular matrix organization” and enhanced “RRNA modification in the nucleous and cytosol”, the latter associated with an activation of p53-mediated nucleolar stress response. Interestingly, specific inhibition of RPS6, RPS2 and RPS9 within this pathway is suggestive of an overall decrease of 40 S ribosome biogenesis, ultimately triggering the activation of p53 [115–117].

The efficacy of the combination metformin/dietary restriction in several cancer types has been also evaluated [77, 118, 119]. In strong concordance, 15 parameters analyzed (out of the 31) were statistically altered in NR-M over NR alone. These included further reduction of cellular proliferation, strong dysregulation of cell cycle progression, and further reduction of all indicators of mitochondrial impairments (Fig. 5) when compared to metformin alone. Molecularly, the proteome of NR-M treated cells reflected a strong activation of intrinsic pathways for apoptosis and TP53 genes, in line with previous reports [9]. Herein, we also identify an upregulation of the tRNA aminoacylation processes which could be associated with cellular stress and energy availability [120].

Finally, we addressed whether fasting was able to further expand the synergistic effect of the S:M co-treatment. Strikingly, addition of NR to the cocktail S:M reached the maximum effectiveness of all interventions tested, impacting ∼78% of the parameters analyzed (24 out of 31) when compared to AL control, and ∼35% (11 out of 30) when compared to dual S:M therapy. These included the highest reduction of cellular proliferation, the highest levels of early and late apoptosis, and the highest retention in SubG1 phase, suggesting the presence of DNA fragments mediated by cellular apoptosis. Likewise, high levels of cellular S phase, in which cells are unable to duplicate its DNA, concomitant with a reduction of CDK2 expression, which are considered associated mechanisms for cellular arrest and apoptosis induction, respectively [121, 122], were cellular features observed under NR-S:M condition. Of relevance, our polytherapy intervention blunted both mitochondrial flexibility (exhibiting 10- and 16-fold reduction of maximal respiration and spare respiratory capacity, respectively), and glycolytic plasticity (exhibiting a 4-, 2.5-, and 45- fold reduction of basal respiration, glycolytic capacity, and glycolytic reserve, respectively). Thus, the potency of the intervention at preventing HCC expansion seems explicable since HCC is frequently associated with enhanced glycolysis, increased expression of glycolytic enzymes, and increase of mitochondrial function [123]. In our study, strong diminution of key energetic enzymes involved in the Glycolytic, Gluconeogenic, and Glycogenolytic pathways contributed to the cellular metabolic crisis. Consistent with this, we uncovered a specific proteomic signature of 67 proteins with significant implication in “Cellular response to stress” where are included key pathways such as “Apoptotic execution phase”, “Programmed Cell death”, “Autophagy”, “Transcriptional Regulation of TP53”, among others. Furthermore, we identified a molecular gene subset (Top10 distinctive proteins) with robust predictive value for overall poor prognosis and survival. One notable example is UGP2 (uridine diphosphate (UDP)-glucose pyrophosphorylase 2), which was found upregulated by NR-S:M conditions. UGP2 exhibits a consistently reduced expression in various cohorts of liver cancer patients, and the lower levels emerge as a significant prognostic factor in these cohorts [124, 125]. On the other hand, MRPL17, MDIG, GIPC1, NAGPA and FAM127A were found downregulated in NR-S:M conditions. MRPL17 participates in the assembly and functioning of the mitochondrial ribosome and has been characterized as a biomarker for HCC diagnosis [126]. MDIG is an oncogenic factor that promotes cell migration, cell-cycle transition, and cellular proliferation in HCC and other cancer types [127]. GIPC1, also known as synectin, constitutes a scaffolding protein that regulates cell surface receptor expression and trafficking. By regulating PDGFR, GIPC1 promotes fibrogenesis and liver disease [128]. NAGPA was found upregulated in liver cancer patients and was associated with poor prognosis [129]. Finally, FAM127A (also known as RTLC8) was found overexpressed in mesenchymal cells from extrahepatic cholangiocarcinoma [130]. Despite this study, the role of FAM127A in HCC have not been explored yet.

In sum, our results support the notion that integration of glucose-limiting strategies (NR) with OXPHOS inhibition (Sorafenib and Metformin) represent attractive therapeutic tools that deserve further investigation for the treatment of liver tumor pathology. In our in vitro experiments, we demonstrate that introducing NR to S:M co-treatment permits a reduction in chemotherapy doses and reprograms liver cancer cells to a bioenergetic status indicative of energetic collapse. Ultimately, this process blunts cellular and metabolic plasticity promoting cellular death.

## Electronic supplementary material

Below is the link to the electronic supplementary material.

Supplementary Material 1

Supplementary Material 2

Supplementary Material 3

Supplementary Material 4

Supplementary Material 5

Supplementary Material 6

## Data Availability

The authors confirm that the data supporting the findings of this study are available within the article and its supplementary materials.

## Abbreviations

AEs: Adverse effects
AL: Ad libitum
ATP-LR: ATP-linked respiration
BsG: Basal glycolysis
BsR: Basal respiration
CDI: Coefficient of drug interaction
CR: Caloric restriction
DEPs: Differential expressed proteins
ECAR: Extracellular acidification rate
G: Glycolysis
GC: Glycolytic capacity
GR: Glycolytic reserve
HBV: Hepatitis B virus
HCC: Hepatocellular carcinoma
HCV: Hepatitis C virus
MTT: Methylthiazolyldiphenyl-tetrazolium bromide
MxR: Maximal respiration
NAFLD: Nonalcoholic fatty liver disease
NASH: Nonalcoholic steatohepatitis
nGA: Non-glycolityc acidification
NR-S:M: Nutrient restriction + S:M
NR: Nutrient restriction
NTAT: Non-tumoral adjacent tumor
OCR: Oxygen consumption rate
PDGFR-β: Platelet-derived growth factor receptor β
S:M: Sorafenib:Metformin
SRC: Spare respiratory capacity
T2DM: Type 2 diabetes mellitus
VEGFRs: Vascular endothelial growth factor receptors
