# Supplementary figures and images for: Fasting in combination with the cocktail Sorafenib:Metformin blunts cellular plasticity and promotes liver cancer cell death via poly-metabolic exhaustion
Source: Cell Oncol (Dordr). 2024 Jul 11;48(1):161–82. doi: 10.1007/s13402-024-00966-2 (PMC11850423; doi:10.1007/s13402-024-00966-2)

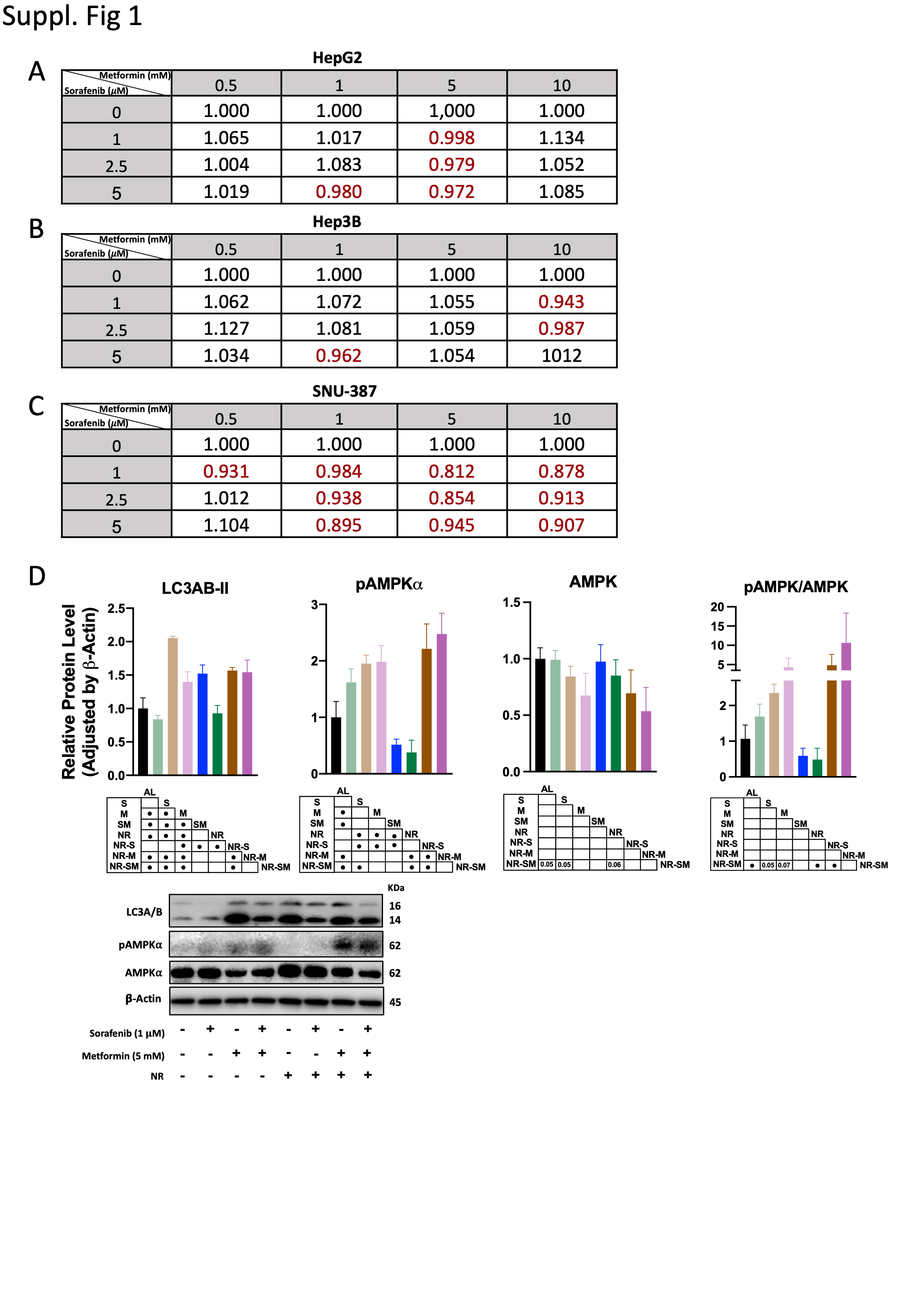

Supplement: Supplementary file 2 — Supplementary Material 2 [file 13402_2024_966_MOESM2_ESM.tiff]

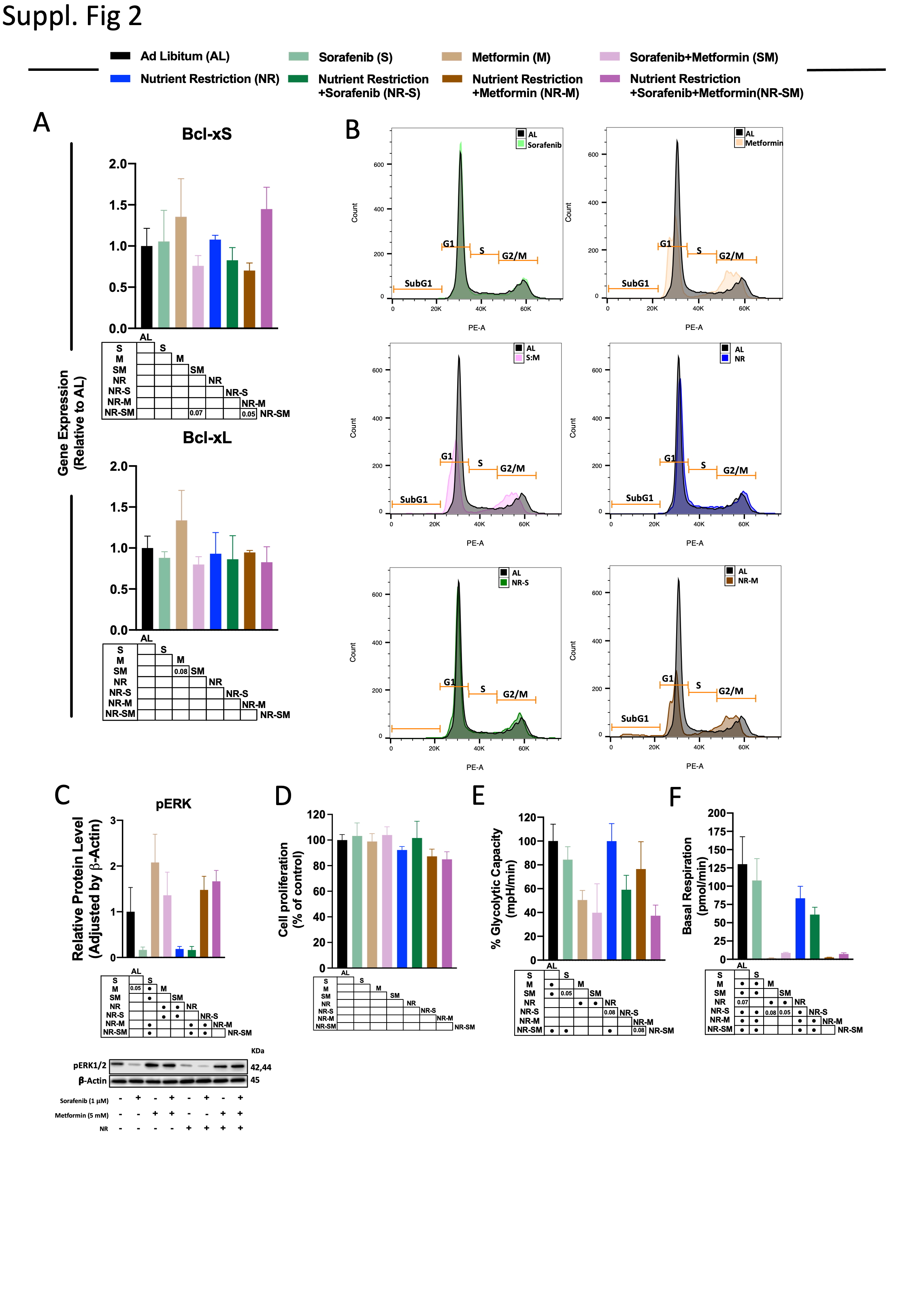

Supplement: Supplementary file 3 — Supplementary Material 3 [file 13402_2024_966_MOESM3_ESM.tiff]

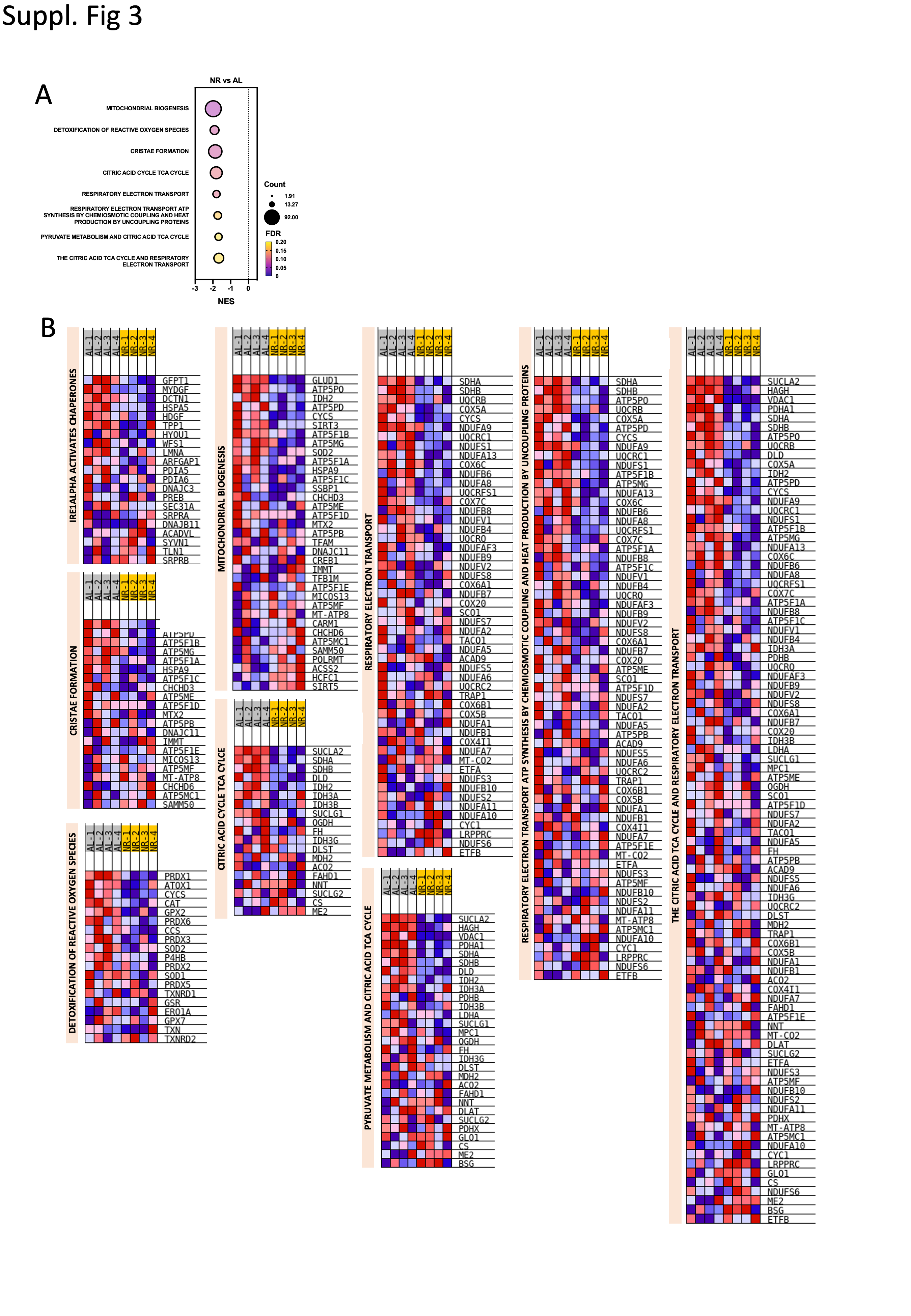

Supplement: Supplementary file 4 — Supplementary Material 4 [file 13402_2024_966_MOESM4_ESM.tiff]

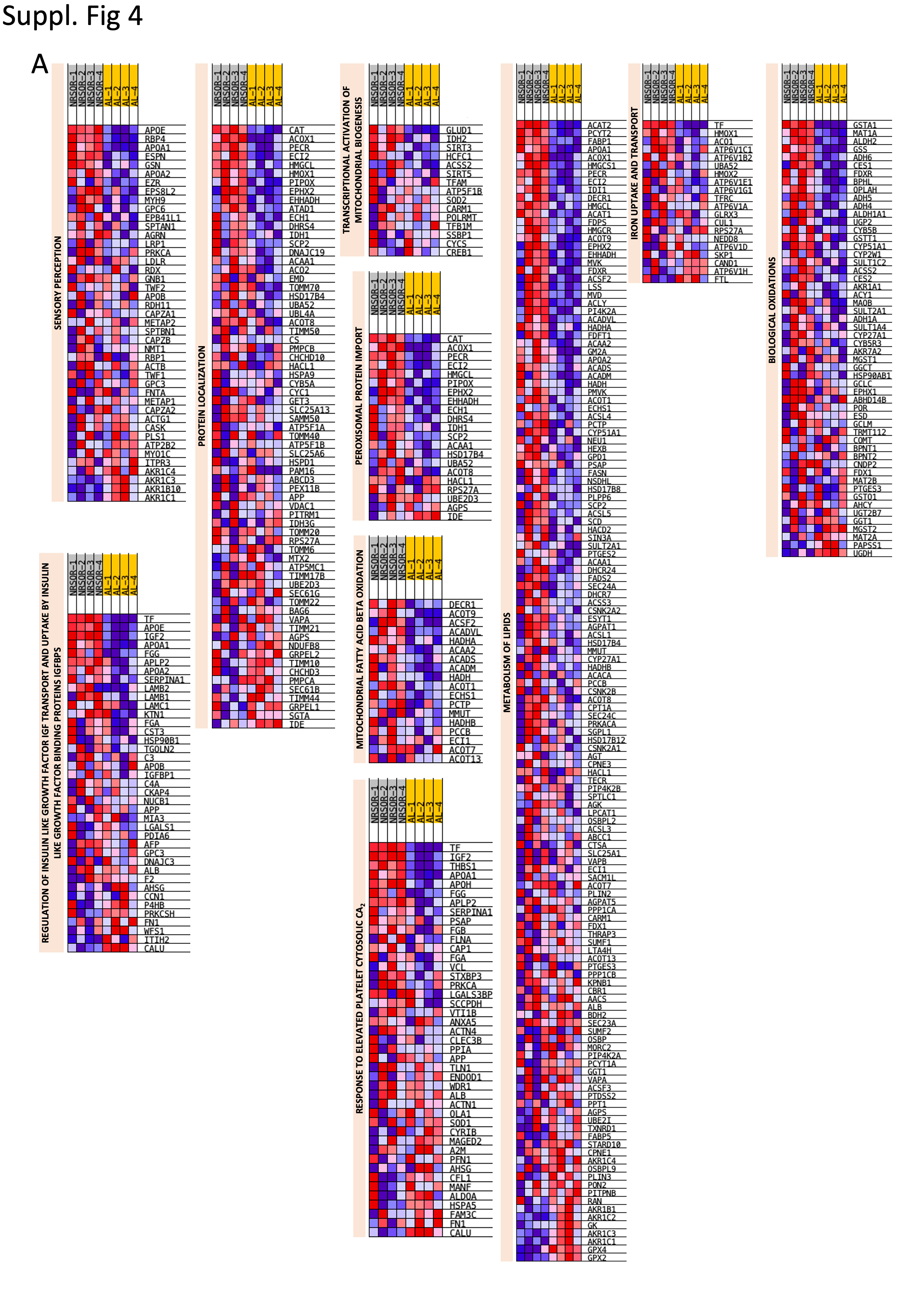

Supplement: Supplementary file 5 — Supplementary Material 5 [file 13402_2024_966_MOESM5_ESM.tiff]
