# Supplementary material for: Fasting in combination with the cocktail Sorafenib:Metformin blunts cellular plasticity and promotes liver cancer cell death via poly-metabolic exhaustion
Source: Cell Oncol (Dordr). 2024 Jul 11;48(1):161–82. doi: 10.1007/s13402-024-00966-2 (PMC11850423; doi:10.1007/s13402-024-00966-2)
Supplement: Supplementary file 6 — Supplementary Material 6 [file 13402_2024_966_MOESM6_ESM.docx]

**Supplementary Figure Legends**

**Suppl. Figure 1. Synergistic antiproliferative effect calculated by the coefficient of drug interaction (CDI).** (A-C) CDI values obtained by cell proliferation in response of co-treatment of Sorafenib (1-5 μM) and Metformin (0,5-10 mM) in HepG2, Hep3B and SNU-387 cell lines in AL conditions. Red: Synergistic antiproliferative effect. (D) Relative abundances of LC3A/B, phosphor-AMPKα, AMPKα and Ratio phosphor-AMPKα/AMPKα protein in response of co-treatment S:M (1 μM-5 mM) in AL and NR conditions compared to non-treated AL cells. The data are presented as the means ± SEMs. The point in table, indicate statistically significant differences (• p<0,05).

**Suppl. Figure 2. Apoptosis, Cell Cycle, Basal Respiration and Glycolytic capacity in response to NR-S:M.** (A) Relative expression at mRNA of *Bcl-xS* and *Bcl-xL* in response of co-treatment S:M (1 μM -5 mM) in AL and NR conditions compared to non-treated AL cells. (B) Representative cell cycle panels in response of Sorafenib, Metformin, S:M, NR, NR-S and NR-M vs. AL. (C) Relative abundances of phospho-ERK1/2 protein in response of co-treatment S:M (1 μM -5 mM) in AL and NR conditions compared to non-treated AL cells. (D) Cell proliferation of HepG2 cells in response of 3hrs. of co-treatment S:M(1 μM-5 mM) in AL and NR conditions compared to non-treated AL cells. (E) % Glycolytic capacity (from ECAR) in response of co-treatment S:M (1 μM-5 mM) in AL and NR conditions compared to non-treated AL cells. (F) Basal Respiration (from OCR) in response of co-treatment S:M (1 μM-5 mM) in AL and NR conditions compared to non-treated AL cells. The data are presented as the means ± SEMs. The point in table, indicate statistically significant differences (• p<0,05).

**Suppl. Figure 3.** **Enrichment pathways from NR vs AL**. (A) Pathways reported by GSEA from NR vs AL conditions. (B) Heatmaps from individual pathway from NR vs AL conditions.

**Suppl. Figure 4.** **Enrichment pathways from NR-S vs AL** (A) Heatmaps from individual pathway from NR-S vs AL conditions.
